# Redescription and neotype designation of *Dopasia formosensis* (Kishida, 1930) (Squamata, Anguidae) from Taiwan

**DOI:** 10.3897/zookeys.1270.173752

**Published:** 2026-02-19

**Authors:** Si-Min Lin, Chin-Chia Shen, Te-En Lin, Yu-Jhen Liang, Wen-Hsien Chang

**Affiliations:** 1 School of Life Science, National Taiwan Normal University, Taipei, Taiwan National Taiwan Normal University Taipei Taiwan; 2 Taiwan Biodiversity Research Institute, Ministry of Agriculture, Nantou, Taiwan Taiwan Biodiversity Research Institute, Ministry of Agriculture Nantou Taiwan; 3 Taipei Zoo, Taipei, Taiwan Taipei Zoo Taipei Taiwan; 4 Vivarium Ecological Engineering Co., Ltd., New Taipei City, Taiwan Vivarium Ecological Engineering Co., Ltd. New Taipei City Taiwan

**Keywords:** *Dopasia harti*, *Dopasia hainanensis*, neotype, parental care, sexual dimorphism, Taiwan

## Abstract

The legless anguid lizard *Dopasia* Gray, 1853 is one of the most secretive and least studied lizard groups. Historically, two *Dopasia* (Squamata: Anguidae) names have been applied to Taiwanese material: *D. harti* (Boulenger, 1899), originally described from Fujian, China and characterized by conspicuous bluish dorsal blotches, and *D. formosensis* (Kishida, 1930), described from Taiwan and reported to lack such markings. Taiwanese specimens were long treated as representing two species until Lin et al. (2003) synonymized *D. formosensis* with *D. harti* after finding no consistent molecular or scalation differences, and interpreted the presence/absence of bluish blotches as sexual dichromatism. Because topotypic *D. harti* was not examined in that study and the original type material of *D. formosensis* has been lost, the application of these names to Taiwanese populations has remained uncertain. Here, we designate a neotype for *D. formosensis* and provide a detailed redescription based on vouchered specimens from Taiwan. We present standardized morphometric and meristic datasets, including X-ray–based vertebral counts, and generate new mitochondrial sequences to document genetic variation. With the aim of facilitating repeatable comparisons within the East Asian *Dopasia* species complex, all measurements and counts are made openly available to support future integrative taxonomic reassessments and to improve nomenclatural stability for this rarely encountered lineage. Taken together with currently available lines of evidence, these results suggest recognizing *Dopasia formosensis* (Kishida, 1930) as a distinct evolutionary lineage, pending a formal systematic revision of the genus.

## Introduction

Among the Squamata taxa known from Taiwan, *Dopasia* Gray, 1853 is one of the least studied groups (Lin e al. 2003). These legless lizards are generally secretive, living under leaf litter and humus in moist forests, which makes field observations and ecological studies extremely difficult (Jablonski et al. 2020). Consequently, knowledge of their natural history, geographic distribution, and evolutionary distinctiveness remains limited across Taiwan and East Asia (Lin e al. 2003; Jablonski et al. 2020).

**Figure 1.**
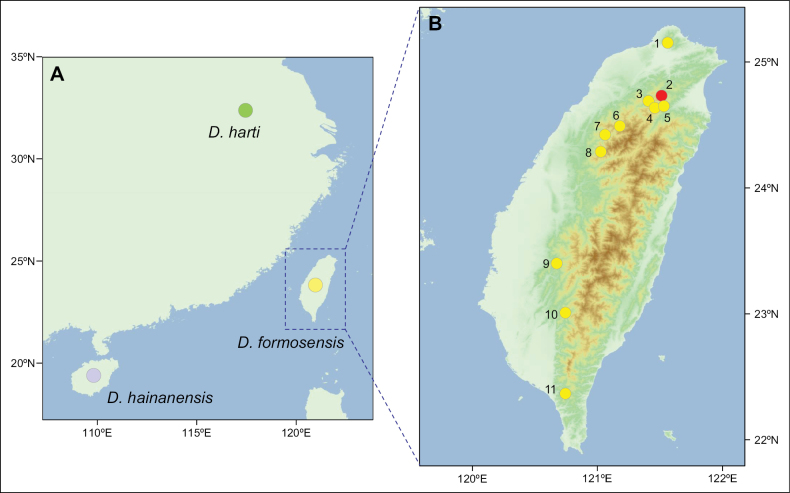
**A**. Sampling sites of *Dopasia formosensis* (Kishida, 1930), *D. harti* (Boulenger, 1899), and *D. hainanensis* (Yang, 1983) available from GenBank; **B**. Sample localities in this study: 1 = Yangmingshan, Taipei; 2 = Hinokiyama (type locality in 1930); 3 = Baling, Taoyuan; 4 = Siling, Taoyuan; 5 = Mingchi, Yilan (sampling site of the neotype); 6 = Guanwu, Miaoli; 7 = Xuejian, Miaoli; 8 = Dahsuehshan, Taichung; 9 = Zhongpu, Jiayi; 10 = Tengzhi, Kaohsiung; 11 = Dahanshan, Pingtung.

The taxonomic status of *Dopasia* in Taiwan has long been a subject of controversy. The first *Dopasia* specimen from Taiwan, originally classified under *Ophisaurus* prior to the genus-level redefinition (Conrad and Norell 2008; Conrad et al. 2010), was reported by Van Denburgh (1909) based on material collected from Tamsui by the renowned Canadian missionary-naturalist Dr. George Leslie Mackay. At that time, the broader definition of “Tamsui” encompassed the Yangmingshan area, which is now known as a relatively stable distribution range for *Dopasia* (Fig. 1), and also the likely locality where the specimen was collected. Van Denburgh initially identified the specimen as *Ophisaurus harti* Boulenger, 1899, but did not exclude the possibility that it might represent a distinct species. Subsequently, Stejneger (1910) listed the name as “undetermined” in his inventory of Taiwan’s herpetofauna. However, after examining specimens from both the type locality (Fuzhou, China) and Taiwan, he concluded that the Taiwanese specimens indeed represented *O. harti* (Stejneger, 1919).

*Ophisaurus formosensis* Kishida, 1930, was later described by Kyukichi Kishida as the second species. The holotype is an adult female collected from “Hinokiyama, Bunzangori, Taihoku-shiu”, meaning the “Hinoki Mountain” near the Taipei City. The type location is ~ 1,400 meters in elevation in what is now the Fuba Cross-ridge Trail (Fig. 1). Kishida distinguished *O. formosensis* from *O. harti* primarily on three traits: (1) absence of bluish dorsal markings; (2) absence of black speckling on the head; and (3) a fan-shaped occipital scale sharply contrasting with adjacent scales. However, the latter two characters have not been referenced in subsequent literature. For over 70 years from 1930 to 2003, it was widely believed that two species of *Dopasia* co-occurred in Taiwan—individuals with bluish dorsal markings identified as *D. harti*, and those lacking such markings as *D. formosensis*. The latter was considered much rarer.

Since 1930, at least five additional diagnostic traits have been proposed, though often adding confusion rather than clarity (organized in Lin et al. 2003). The most frequently cited has been the number of dorsal scale rows between the lateral folds: *D. formosensis* supposedly with 14 rows, and *D. harti* with 16–18. This character has been mentioned in numerous publications since first proposed by Liu-Yu (1970), and followed by Wang and Liang (1976) and Chen and Yu (1984). However, specimens preserved in alcohol or formalin often exhibit compressed lateral folds, making accurate scale counts unreliable.

In the 2003 study, Lin et al. combined mitochondrial DNA analysis (12S rRNA and cytochrome *b*) with detailed morphological comparisons included both “*D. harti*” with blue dorsal markings and “*D. formosensis*” without markings. The results demonstrated that multiple haplotypes were shared between the two forms, and phylogenetic analyses showed no genetic differentiation between the two. Morphological evaluation further demonstrated that characters such as head scalation and dorsal scale row numbers were either inconsistently defined in different literature, or altered by preservation artifacts. Based on these findings, Lin et al. (2003) concluded that both forms represented a single species in Taiwan, with coloration differences most plausibly explained by sexual dichromatism. Females and some hatchlings tend to lack bluish markings, while adult males exhibit them, suggesting a role in secondary sexual signaling. They therefore recommended treating *D. formosensis* as a junior synonym of *D. harti*, while leaving open the possibility of future reevaluation if stronger evidence for differentiation emerged.

With growing academic interest in legless lizards, researchers in Asia have begun to re-examine this historically confusing group. For example, Lavin and Girman (2019) reported 7.0%–8.8% mitochondrial sequence divergence among members of the *D. harti*, *D. formosensis*, and *D. hainanensis* complex, suggesting the potential validity of these allopatric taxa as distinct species. In contrast, Li et al. (2024) estimated a p-distance of 0.0815 between *D. formosensis* and *D. harti*, and 0.0709 between *D. formosensis* and *D. hainanensis*, values comparable to those reported by Lavin and Girman (2019). However, due to the lack of morphological differentiation, Li et al. (2024) treated both *D. formosensis* and *D. hainanensis* as junior synonyms of *D. harti*. Almost at the same time, Rao (2024) recognized all three taxa as valid species in his comprehensive field guide to the lizards of China. These differing viewpoints indicated the urgent need for integrative taxonomic studies that combine molecular evidence with detailed morphological analyses.

The taxonomic status of *D. formosensis* remains unresolved primarily due to the lack of sufficient sampling for morphological analyses, and the loss of its holotype. This situation further highlights the importance of designating a neotype supported by robust genetic and morphological data to stabilize the taxonomy of this group. In this study, we aim to help clarifying the taxonomic status of Taiwanese *Dopasia* by: (1) generating new DNA sequences and comparing them against available *Dopasia* lineages adjacent Asian region; (2) designating a neotype for *D. formosensis* and providing detailed morphological descriptions of the neotype and other topotypes; and (3) making these datasets publicly available to facilitate further comparative research across East Asia. Through these efforts, we aim to provide a more stable taxonomic framework for future ecological and conservation studies of this overlooked lizard group.

## Materials and methods

### Samples

Due to the unpredictable occurrence of *Dopasia* within its natural range, and its current status as a protected species in Taiwan, no new specimens were collected for this study. Instead, we utilized specimens preserved in the Department of Life Science, National Taiwan Normal University from 1998 to 2003 for both morphological and genetic analyses. As these specimens are scheduled to be donated to the National Museum of Natural Science (**NMNS**), their catalog numbers are designated with the NMNS prefix. In addition, we used genetic tissue samples (TBRI RN series) collected approximately between 2010 and 2020 through the Taiwan Roadkill Observation Network, a citizen science platform maintained by the Taiwan Biodiversity Research Institute. Detailed specimen information is provided in Fig. 1, Table 1. Treatment and handling of this protected species were approved by Forestry and Nature Conservation Agency, Ministry of Agriculture, Taiwan (No. 1140241951).

**Table 1.** Morphological specimens including the type series, and genetic samples with GenBank accession numbers used in this study.

Voucher ID	Type series	12S	16S	COI	ND4	cytb	Locality	Reference
*D. formosensis*								
NMNS14488	Neotype	N/A	PX229999	PX229448	PX236186	N/A	Mingchi, Yilan, Taiwan	This study
NMNS14489	Other material	N/A	N/A	PX229446	N/A	N/A	Mingchi, Yilan, Taiwan	This study
NMNS14490	Other material	N/A	PX230000	PX229447	PX236185	N/A	Mingchi, Yilan, Taiwan	This study
NMNS14491	Other material	AF380948	PX230001	PX229445	N/A	AF380962	Yangmingshan, Taipei, Taiwan	This study; Lin et al. 2003
NMNS14492	Other material	AF380947	PX230003	PX229439	N/A	AF380956	Guanwu, Miaoli, Taiwan	This study; Lin et al. 2003
NMNS14493	Other material	AF380949	PX229997	PX229451	PX236188	AF380957	Yangmingshan, Taipei, Taiwan	This study; Lin et al. 2003
NMNS14494	Other material	AF380947	PX230002	PX229444	N/A	AF380958	Siji, Yilan, Taiwan	This study; Lin et al. 2003
NMNS14495	Other material						Fushan, New Taipei, Taiwan	This study
NMNS14496	Other material						Chatienshan, New Taipei, Taiwan	This study
NMNS14497	Other material						Yangmingshan, Taipei, Taiwan	This study
NMNS14498	Other material						UN	This study
NMNS14500	Other material						Yangmingshan, Taipei, Taiwan	This study
NMNS14501	Other material						Yangmingshan, Taipei, Taiwan	This study
NMNS14504	Genetics	N/A	N/A	PX229450	PX236187	N/A	Xuejian, Miaoli, Taiwan	This study
NMNS14505	Other material	N/A	PX229998	PX229449	N/A	N/A	Northern Cross-island Highway*	This study
TBRI RN0070	Genetics	N/A	PX230010	N/A	N/A	N/A	Zhongpu, Jiayi, Taiwan	This study
TBRI RN0157	Genetics	N/A	PX230009	PX229440	PX236181	N/A	Yangmingshan, Taipei, Taiwan	This study
TBRI RN0518	Genetics	N/A	PX230005	PX229443	PX236183	N/A	Dahsuehshan, Taichung, Taiwan	This study
TBRI RN0588	Genetics	N/A	PX230011	PX229441	N/A	N/A	Siling, Taoyuan, Taiwan	This study
TBRI RN0904	Genetics	N/A	PX230008	N/A	N/A	N/A	Tengzhi, Kaohsiung, Taiwan	This study
TBRI RN1812	Genetics	N/A	PX230006	PX229442	PX236182	N/A	Baling, Taoyuan, Taiwan	This study
TBRI RN1900	Genetics	N/A	PX230007	N/A	N/A	N/A	Dahanshan, Pingdong, Taiwan	This study
TBRI RN2851	Genetics	N/A	PX230004	N/A	PX236184	N/A	Siling, Taoyuan, Taiwan	This study
*D. harti*								
N/A		KF279681	KF279681	KF279681	KF279681	KF279681	Huangshan, Anhui, China	Pan et al. 2015
N/A		KF806482	KF806482	KF806482	KF806482	KF806482	China	Yang et al. 2015, DS
*D. hainanensis*								
CB2018037		MN640999	MN640999	MN640999	MN640999	MN640999	Hainan Island, China	Cai et al. 2020
CTMZ-04924		N/A	N/A	MW021411	N/A	N/A	N/A	Che et al. 2021, DS
KIZ014749		N/A	N/A	MW021343	N/A	N/A	N/A	Che et al. 2021, DS
KIZYPX19170		N/A	N/A	MW021410	N/A	N/A	N/A	Che et al. 2021, DS
*D. gracilis*								
N/A		KU885977	KU885977	KU885977	KU885977	KU885977	Tibetan Plateau, China	Fang 2016, DS
RE13001		KJ941042	KJ941042	KJ941042	KJ941042	KJ941042	Gejiu City, Yunnan Province, China	Yan et al. 2016, DS
AMNH15377		N/A	MK107677	N/A	MK107795	N/A	Ha Gaing, Vietnam	Lavin and Girman 2019
CAS233231		N/A	MK107655	N/A	MK107773	N/A	Chin State, Myanmar	Lavin and Girman 2019
ROM40319		N/A	MK107660	N/A	MK107778	N/A	Lao Cai, Vietnam	Lavin and Girman 2019
ROM40320		N/A	MK107661	N/A	MK107779	N/A	Lao Cai, Vietnam	Lavin and Girman 2019
ML01		MN661343	MN661343	MN661343	MN661343	MN661343	Mile city, Yunnan province, China	Cai et al. 2020
N/A		PQ505668	PQ505668	PQ505668	PQ505668	PQ505668	N/A	Luo 2024, DS
N/A		PP003927	PP003927	PP003927	PP003927	PP003927	Baoshan, Yunnan, China	Zhan et al. 2024
*D. sokolovi*								
NCSM77336		N/A	MK107662	N/A	MK107780	MK107690	Lam Dong, Vietnam	Lavin and Girman 2019
*Dopasia* sp.								
MVZ 224111		N/A	MK107657	N/A	MK107775	MK107687	Vinh Phuc, Vietnam	Lavin and Girman 2019
MVZ 230055		N/A	MK107658	N/A	MK107776	MK107688	Vinh Phuc, Vietnam	Lavin and Girman 2019
*O. attenuatus*								
N/A		EU747729	EU747729	EU747729	EU747729	EU747729	United States	Castoe et al. 2008

*: Northern Cross-island Highway could be either Baling, Siling, or Mingchi which are closely located from each other. Therefore, some roadkill collector did not distinguish these localities.

### DNA sequencing and genetic comparison

In order to evaluate the genetic differentiation between Taiwanese and other Asian samples, we sequenced three mitochondrial fragments including 16S rDNA, cytochrome *c* oxidase I (COI), and NADH dehydrogenase subunit 4 (ND4). DNA extraction was conducted using EasyPure Genomic DNA mini Kit (Bioman, New Taipei). We then amplified the target fragments using three pairs of primers as listed in Table 2. The reactions were performed in a total of 20 μl volume containing 1 μl of DNA, 10 μl of 2× GoTaq® Green Master Mix (Promega, France), 0.2 μl of 10 mM forward and reverse primers, and 0.2–0.5 μl of 2.5 mM MgCl2. The PCR conditions consisted of denaturation at 94 °C for 4 min, followed by 40 cycles of denaturation at 94 °C for 30 sec, annealing at suggested T_m_ (Table 2) for 45 sec, and extension at 72 °C for 60 sec, with a final extension at 72 °C for 10 min. All PCR processes were performed by using a Biometra TOne Thermal Cycler (Analytic Jena, Germany). Electrophoresis was performed to assess the quality of PCR products on 1.2% agarose TBE gel, which was stained by FloroStain DNA Florescent Staining Dye (SMOBio, Taiwan).

**Table 2.** Primers and suggested annealing temperatures (T_m_) used for PCR amplification in this study.

Gene	Primer	Sequence	T_m_	Reference
12S	PL1	AGTCTGCTCAAAAAGATTAATGTTAA	55 °C	Lin et al. 2003
	PH1	TCTTGGTCTGAAACCTCAGTTACCTA		
16S	16SAR	CGCCTGTTTAYCAAAAACAT	55 °C	This study
	16SBR	CCGGTYTGAACTCAGATCAYGT		
COXI	ChmF4	TYTCWACWAAYCAYAAAGAYATCGG	55 °C	This study
	ChmR4	ACYTCRGGRTGRCCRAARAATCA		
ND4	DopND4F	CACCTATGACTTCCGAAAGC	56.2 °C	This study
	DopND4R	AAGGCCATGGGATTACTTCT		
cytochrome *b*	PL2	CCMTCMAACMTYTCMDYWTKRTGAAA	55 °C	Lin et al. 2003
	PH2	GGCRAAKARRAARTAYCATTC		

PCR products were sequenced bidirectionally using an ABI 3730XL autosequencer (Genomics BioSci & Tech Corp., Taipei, Taiwan). Raw sequence data were assembled and edited using Sequencher 5.4.6 (Gene Codes Corporation, Boston, MA, USA) and aligned with the Clustalw (Thompson et al. 1994) implementation in MEGA 11 (Tamura et al. 2021).

### Phylogenetic analyses

We included sequences of published *Dopasia* retrieved from GenBank in our phylogenetic analyses, including *D. harti*, *D. hainanensis*, *D. gracilis*, *D. sokolovi*, and *Ophisaurus attenuatus* as the outgroup (Table 1). In this process, 12S rRNA and cytochrome *b* sequences from our previous study (Lin et al. 2003) were also incorporated in the analyses. All sequences were aligned using Clustalw (Thompson et al. 1994) implementation in MEGA 11 (Tamura et al. 2021). Phylogenetic trees were reconstructed for the concatenated sequences using both maximum likelihood (ML) and Bayesian inference (BI) approaches. ML analyses were conducted in IQ-TREE 2 (Minh et al. 2020) using ModelFinder Plus (Kalyaanamoorthy et al. 2017) to select the best-fit nucleotide substitution models based on the Bayesian information criterion (BIC), which indicated GTR+F as the best-fit model for all the five genes. Branch support was assessed using 10,000 ultrafast bootstrap (UFBoot) replicates (Hoang et al. 2018). For Bayesian analyses, we used MrBayes 3.2.7 (Ronquist et. al. 2012), with substitution models selected using jModelTest 2.1.10 (Posada 2008) based on BIC: HKY+I+G for 18S rDNA, GTR+I for 16S RNA, GTR+G for COI, GTR+I for ND4, and HKY+G for cytochrome *b*. Each MCMC analysis was run for 4,000,000 generations with sampling every 1,000 generations, and the first 25% of trees discarded as burn-in. Convergence was assessed by ensuring that the average standard deviation of split frequencies fell below 0.01. Nodes with UFBoot values ≥ 95 and Bayesian posterior probabilities (PP) ≥ 0.95 were considered as strongly supported.

### Morphology

Morphological traits examined in this study followed Brygoo (1987), Lin et al. (2003), Nguyen et al. (2011), and with further modifications after Kai Wang (pers. comm.) and Martina Lawson & Daniel Jablonski (pers. comm.). All measurements were taken by SML with a Mitutoyo digital caliper to the nearest 0.1 mm. Body measurements were taken for the following traits: total length (**TotL**), from the tip of the rostral scale to the tip of the tail; snout–vent length (**SVL**), from the tip of the rostral scale to the vent; tail length (**TaL**), from the vent to the tip of the tail, only valid when the tail is intact; body width (**BW1**) and body height (**BH1**) at one-head length posterior of neck; body width (**BW2**) and body height (**BH2**) at mid SVL; body width (**BW3**) and body height (**BH3**) at one-head length anterior of cloaca; tail width (**TW**) and tail height (**TH**) at one head-length posterior of cloaca. Head measurements were taken for the following traits: head length (**HL**), from the tip of the snout to the posterior margin of the fan-shaped occipital plate; head width (**HW**), the widest point of the head posterior to the eyes; head height (**HH**), the highest part of the head posterior to the eyes; nostril diameter (**ND**), the diameter of the nostril; eye size (**EyS**), width of the orbit, measured in an anterior-posterior direction; ear opening diameter (**ErD**), the diameter of the ear opening; snout–eye distance (**SEyD**), from the tip of snout to the anterior margin of the orbit; snout–ear distance (**SErD**), from the tip of snout to the anterior margin of the ear opening; snout–fold distance (**SFdD**), from the tip of snout to the anterior margin of the lateral fold; nostril–eye distance (**NEyD**), from nostril to the anterior margin of the orbit; nostril–ear distance (**NErD**), from nostril to the anterior margin of the ear opening; eye–ear distance (**EyErD**), from the posterior margin of the orbit to the anterior margin of the ear opening; ear–fold distance (**ErFD**), from the posterior margin of the ear opening to the anterior margin of lateral fold; mouth length (**MoL**), from the tip of snout to the margin of mouth opening (commissure); mandible length (**MaL**), from the tip of the snout to the posterior extremity of the lower jaw (mandible); snout–frontal distance (**SFtD**), from the tip of snout to the anterior margin of the frontal shield; frontal shield length (**FL**); frontal shield width (**FW**); rostrum width (**RW**); nostril width (**NW**), the transverse distance between the two nostrils; and jaw width (**JW**), the transverse distance between the left and right corners of the mouth. All measurements were taken from the left side of the animal unless the trait was not accessible on that side or other exceptions applied. Such exceptions include cases where certain features could not be measured due to distortion or curvature of the specimen during preservation. In those instances, measurements were taken from the more extended side of the body.

Scale counts were made under a Leica S9D dissecting microscope which was connected to a Leica Flexacam C3 microscope camera system. We acquired scale counts including dorsal scales longitudinal (**DSL**), the number of longitudinal scales from the nuchal scales (but not included) to the dorsum opposite of the vent; ventral scales longitudinal (**VSL**), counted from the scales between the first pair of chin shields (= first gular) to the precloacal scales, gulars included; scales along lateral fold (**SLF**), counted longitudinally from the start to the end of the lateral fold; dorsal scales rows (**DSR**), transverse rows of dorsal scales between lateral folds; ventral scale rows (**VSR**), transverse rows of ventral scales between lateral folds; caudal scale rows (**CR**), counted around the tail at the position of the tenth subcaudal scales; supraocular (**So**); superciliary (**Sc**); supralabial (**SL**); and infralabial (**IL**). Definitions and descriptions of other scales on head utilized are by Gvoždík et al. (2013), Thanou et al. (2021), and Wang et al. (2025).

Finally, X-ray photographs were taken by Dr. Yuan-Peng Kuan, a veterinarian at Bright Exotic Animal Hospital, to determine the number of presacral vertebrae (**VPS**; from the atlas to the remnants of the hind limb bones, following Brygoo 1987 and Nguyen et al. 2011), caudal vertebrae (**VC**, from the remnants of the hind limb bones to tail tip; available only in specimens with an intact tail), and total vertebrae (**VT**; only in specimens with an intact tail).

## Results

### Phylogenetic relationship

**Figure 2.**
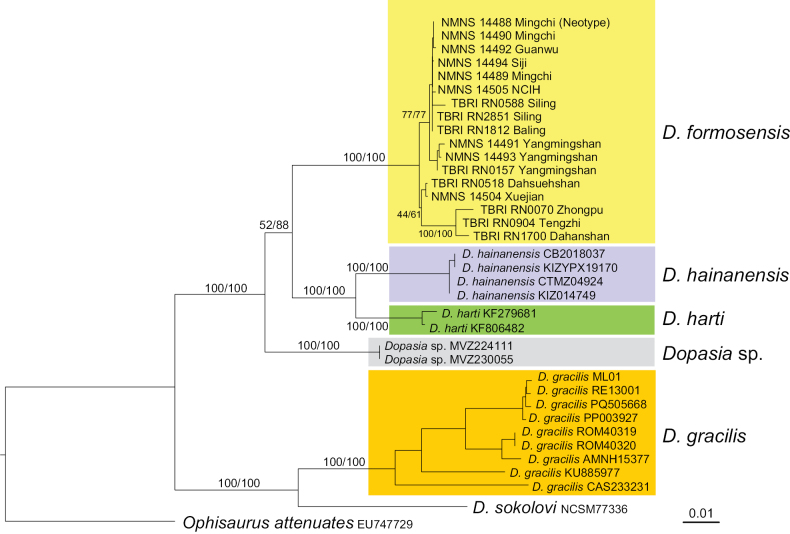
Maximum likelihood tree of *Dopasia formosensis* (Kishida, 1930) compared to other closely related species based on five mitochondrial fragments. Strong genetic divergence was detected among the clades from Taiwan (*D. formosensis*), Hainan (*D. hainanensis*), and continent China (*D. harti*). The values beside the nodes indicate statistical support from 10,000 ultrafast bootstrap replicates and Bayesian posterior probabilities.

Phylogenetic trees reconstructed using both maximum likelihood and Bayesian inference yielded identical topologies (Fig. 2). All Taiwanese sequences formed a well-supported clade with relatively low genetic divergence. The sister clade to the Taiwanese lineage includes *D. hainanensis* and *D. harti*, which together form a distinct monophyletic group. In addition, two individuals, MVZ 224111 and MVZ 230055 originally labeled as *D. hainanensis* and collected from Vietnam in Lavin and Girman (2019), were found to be genetically distinct from other *D. hainanensis* sequences sampled from the species’ type locality. We therefore tentatively label these as *Dopasia* sp. and refrain from assigning them to a species until further taxonomic information is available.

### Morphology evaluation

**Figure 3.**
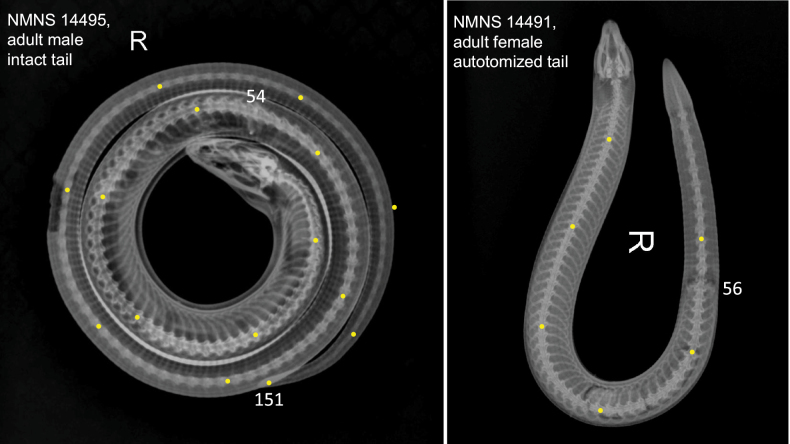
X-ray photographs used to determine the number of presacral vertebrae (VPS; from the atlas to the remnants of the hind limb bones), caudal vertebrae (VC; available only in specimens with an intact tail), and total vertebrae (VT; intact tail only). **A**. NMNS 14495, adult male with intact tail (VPS = 54, VT = 151); **B**. NMNS 14491, adult female with an autotomized tail (VPS = 56). Yellow dots mark every tenth vertebra to facilitate visualization of the counting procedure. Photographed by Dr. Yuan-Peng Kuan, Bright Exotic Animal Hospital.

Detailed measurements and scale counts are provided in Suppl. material 1 and are summarized in a simplified format in Table 3. Vertebral counts were obtained from high-resolution X-ray images as illustrated in Fig. 3, which are available for reference in Suppl. material 2. Summary statistics (including mean, standard deviation, minimum, and maximum values) for morphometric measurements, body proportions, head proportions, scale counts, and vertebral counts are also presented in Table 3.

**Table 3.** Major measurements, relative length, and scale counts of *Dopasia formosensis* in this study.

	Mean	SD	Range
Measurements			
TotL (intact tail)	570.6	48.8	505–629
SVL (adults)	202.7	22.8	171–231
SVL (adult males)	203.3	22.0	175–230
SVL (adult females)	201.3	30.0	171–231
Body ratios			
TaL/SVL	1.828	0.088	1.738–1.953
TaL/TotL	0.646	0.011	0.635–0.661
HL/SVL	0.100	0.009	0.089–0.119
HL/SVL (males)	0.104	0.009	0.092–0.119
HL/SVL (females)	0.097	0.007	0.089–0.102
SErD/SVL	0.115	0.010	0.098–0.135
ErFD/SVL	0.079	0.010	0.065–0.103
SFD/SVL	0.187	0.019	0.161–0.233
Head ratios			
HW/HL	0.721	0.033	0.649–0.771
HH/HL	0.590	0.049	0.512–0.695
EyS/HL	0.165	0.015	0.135–0.194
MouL/HL	0.938	0.042	0.868–1.026
JW/HL	0.666	0.050	0.558–0.749
JW/HL (males)	0.676	0.031	0.633–0.731
JW/HL (females)	0.630	0.067	0.558–0.690
Scale counts			
DSL	119.3	2.1	117–123
VSL	124.9	3.0	120–130
LFS	99.2	1.3	97–101
DSR	15.1	1.5	14–18
VSR	10	0	10
CR	23.1	1.0	22–24
So	4.9	0.3	4–5
Sc	4.9	0.5	4–6
SL	10.7	0.6	10–12
IL	11.5	0.9	10–13
Vertebrae counts			
VPS	54.8	0.8	54–56
VC (intact tail)	95.4	2.3	92–97
VT (intact tail)	150.0	2.9	146–153

Sample sizes: *n* = 6 for intact tail; *n* = 5 for adult males; *n* = 3 for adult females; *n* = 14 for the others.

## Discussion

Since first described in the early 20^th^ century, researchers have debated on whether Taiwan harbors a distinct endemic species, i.e., *D. formosensis* (Kishida, 1930), or whether it simply represents an insular population of the widespread *D. harti* (Boulenger, 1899). This confusion not only complicates biodiversity assessments, but also limits conservation planning. Because Taiwan represents the only known range of *D. formosensis*, we consider it a major contribution of this study to provide careful morphometric measurements and to make these data publicly available, thereby enabling direct genetic and morphological comparisons by future researchers.

With the recent expansion of molecular datasets for Asian anguids, species delimitation within the *D. harti*/*D. formosensis*/*D. hainanensis* complex have been repeatedly revisited. Recent debates include Lin et al. (2003) and Li et al. who (2024) proposed the latter two species as junior synonym of *D. harti*, whereas Lavin and Girman (2019) and Rao (2024) tended to treat them as distinct species. Considering recent studies of squamate diversity in Taiwan and adjacent regions, we suggest that mitochondrial divergence among these three taxa is already comparable to, or even greater than, that observed among many other lizard lineages recognized as distinct species. For example, genetic distances among recently described or proposed Taiwanese lizards include 0.0639–0.0861 in grass lizards (Lue and Lin 2008; Tseng et al. 2015), 0.0654–0.0779 among putative cryptic lineages of agamid lizards (Lin et al. 2025), 0.0400–0.0590 in *Plestiodon* skinks (Kurita et al. 2017), and 0.0722–0.0875 in *Sphenomorphus* skinks (Wang et al. 2025). Given that interspecific divergence within the *Dopasia* complex has been estimated at ~ 0.0700–0.0880 (depending on loci), these values are broadly comparable to divergences reported among recently delimited lizard lineages. Because substitution rates may vary among lineages and loci, we present this comparison only as contextual information rather than as a diagnostic threshold. Taken together with currently available phylogenetic evidence, these results support recognition of *D. formosensis* as a distinct taxon within the complex.

At the same time, interpretations remain sensitive to taxon sampling and to the availability of comparable morphological data. In this context, we provide standardized morphometric and meristic datasets, as well as X-ray–based vertebral counts, to facilitate repeatable cross-study assessments. The present study addresses two previously challenging issues. First, in calculating transverse dorsal scale rows, we excluded the smaller scales adjacent to the lateral fold and counted only the larger, more clearly defined dorsal scales. This adjustment yielded more accurate dorsal scale counts and revealed that a higher proportion of Taiwanese specimens exhibit the “14-row” dorsal scale configuration. While this trait may not serve as a definitive diagnostic character, it nevertheless provides a distinguishable difference from *D. harti*.

Second, to ensure comparability of cephalic characters across studies, we revised the terminology and homology statements for head scalation. Upon re-examination of specimens, we confirmed that all Taiwanese samples possess two suprarostrals posterior to the rostral, followed by a medium-sized frontonasal, a pair of medium-sized prefrontals that may or may not be in medial contact, and a large frontal posteriorly. This definition is congruent with recent treatments of other anguids, which recognize paired prefrontals (either separated or in contact) (Gvoždík et al. 2010; Papenfuss and Parham 2013) and a single frontonasal anterior to the frontal (Gvoždík et al. 2013; Thanou et al. 2021; Li et al. 2024). We hope that this revised definition of head scalation will help clarify past inconsistencies in the literature, which may have originated from Kishida’s (1930) original description. In that work, the pentagonal, enlarged frontonasal was mislabeled as a single prefrontal, and the paired prefrontals were mistakenly regarded as the first supraoculars.

To facilitate comparisons among closely related taxa, Gvoždík et al. (2013) recognized three configurations of prefrontal contact: Type A, in which the paired prefrontals are broadly in contact and separate the frontonasal from the frontal; Type B, in which the prefrontals are either narrowly in contact or separated but arranged in a “four-corner opposition” pattern with only a limited area of medial contact; and Type C, in which the frontonasal and frontal are broadly in contact, clearly separating the paired prefrontals. Based on our observations, all examined specimens of *D. formosensis* correspond to Type B, with the paired prefrontals showing either no or very narrow medial contact and forming the characteristic four-corner opposition pattern.

All measurements and counts in this study are made openly available to enable independent re-evaluation of character variation as additional material becomes available from mainland Asia (*D. harti*) and Hainan (*D. hainanensis*). For example, a preliminary synthesis based on the current dataset (Kai Wang, personal communication) suggests potential morphological differentiation between Taiwanese *D. formosensis* and topotypic *D. harti*, particularly in longer tail proportions (TaL/SVL 173.8–195.3% vs usually < 171%), and slightly higher presacral vertebral counts (VPS 54.8 vs usually < 54). These emerging lines of research indicate that our datasets will likely play an important role in future taxonomic reassessments of this complex.

Compounding this long-standing uncertainty, Kishida’s holotype was apparently lost after World War II, and the absence of a neotype has hindered nomenclatural stability. We therefore designate a neotype and a series of referred specimens and present comprehensive morphological, ecological, and natural history information linked to vouchered material. The justification and procedural details for neotype designation are also provided below.

### Species accounts

#### Dopasia formosensis

Fig. 4

**Figure 4.**
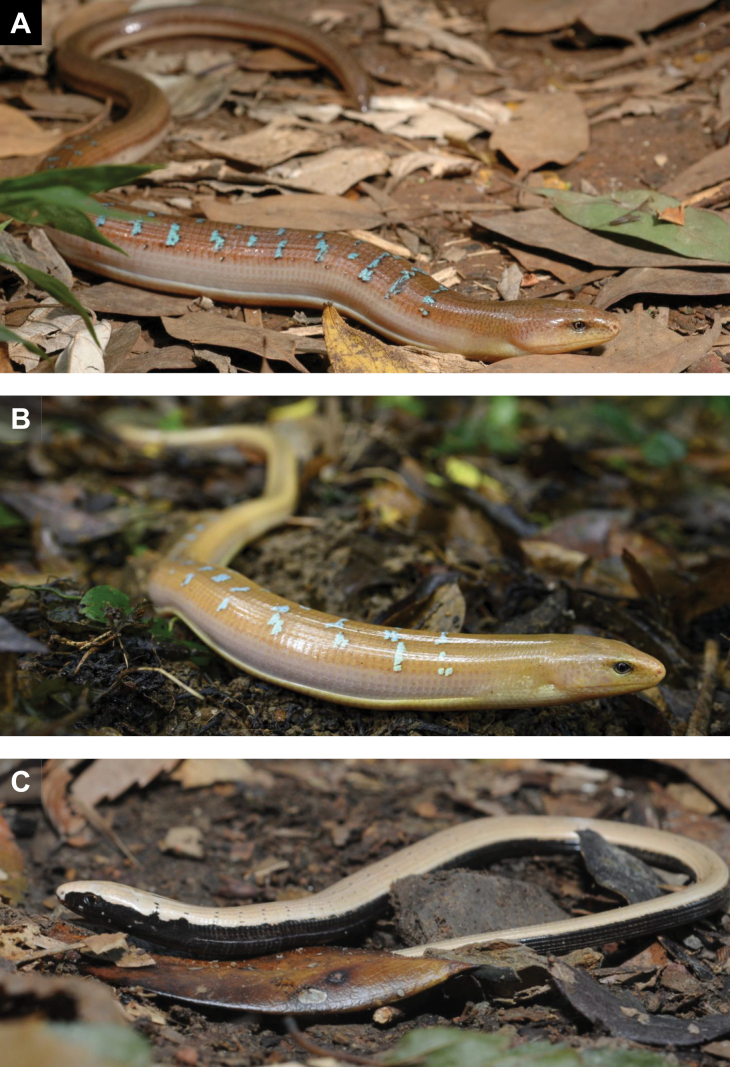
*Dopasia formosensis* in natural habitat. **A**. A fully mature adult male showing its dorsal bluish marking; **B**. A relatively younger male; **C**. A young individual with pale brown dorsal coloration and sharply contrasting black ventral surface. Photographed by Y-JL.

**Chresonymy**. *Ophisaurus harti* — Chen 1956; Wang and Wang 1956; Chen 1969; Liu-Yu 1970; Wang and Liang 1976; Chen and Yu 1984; Lue et al. 1987; Lin and Cheng 1990; Lue 1990; Lue et al. 1999; Shang 2001; Lue et al. 2002; Shang 2007; Shang 2008; Shang et al. 2009.

#### Justification for neotype designation (ICZN Art. 75.3)

**75.3.1. Purpose**. We designate a neotype for *Dopasia formosensis* (Kishida, 1930) with the express purpose of stabilizing and clarifying the taxonomic status and type locality of this nominal species-group taxon.

**75.3.2. Differential diagnosis**. *Dopasia formosensis* is distinguished from topotypic *D. harti* (Fujian) by a suite of characters which were shown below.

**Figure 5.**
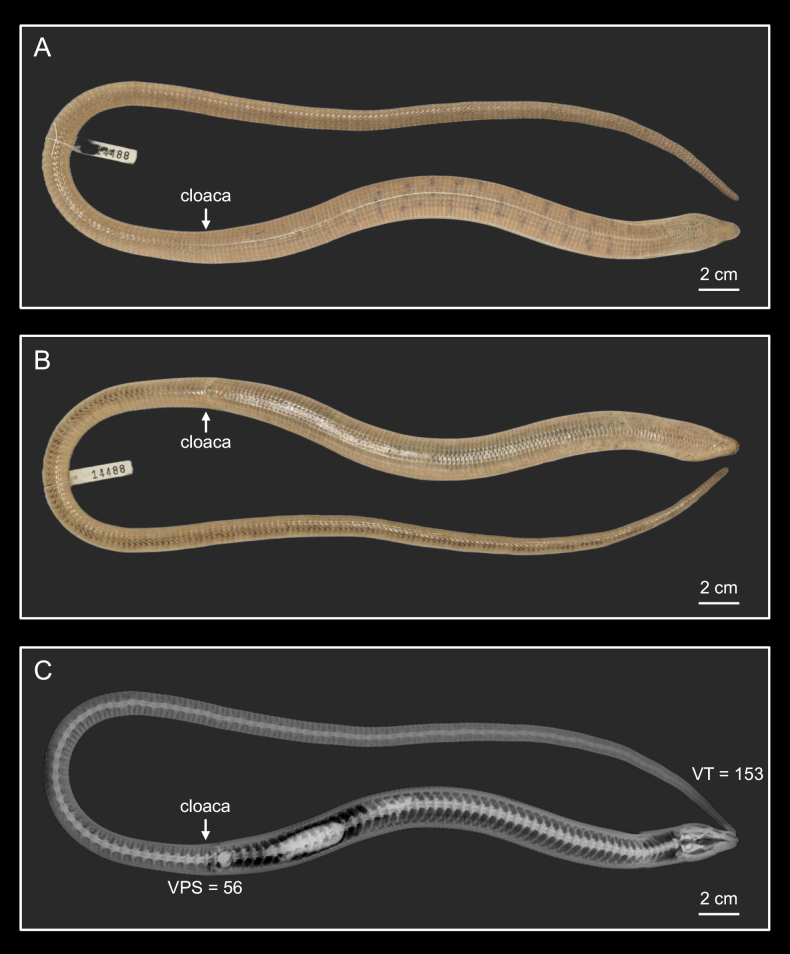
Neotype of *Dopasia formosensis*, NMNS 14488 (SVL = 221 mm, TotL = 605 mm), an adult male from Mingchi, Yilan County, Taiwan. **A**. Dorsal view; **B**. Ventral view; **C**. X-ray image showing presacral and total vertebral counts. Photographed by Chih-Wei Chen, Chin-Chia Shen, and Yuan-Peng Kuan.

**Figure 6.**
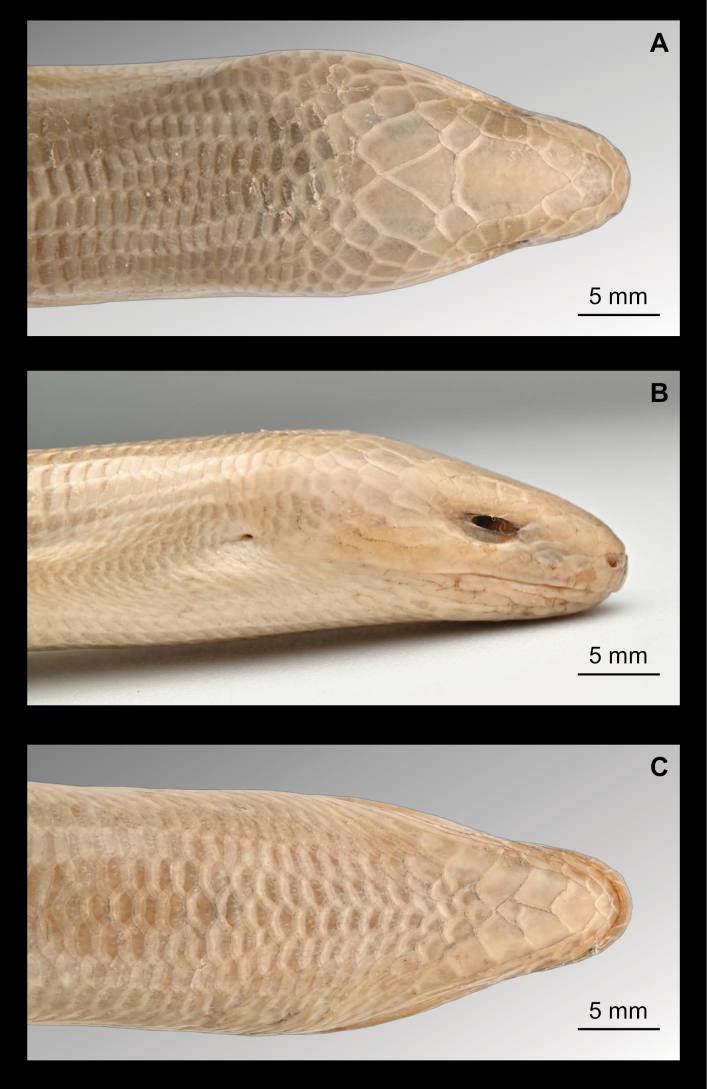
Head morphology of the neotype of *Dopasia formosensis* (NMNS 14488), an adult male from Mingchi, Yilan County, Taiwan. **A**. Dorsal view; **B**. Lateral view; **C**. Ventral view. Photographed by Chih-Wei Chen and Chin-Chia Shen.

**Figure 7.**
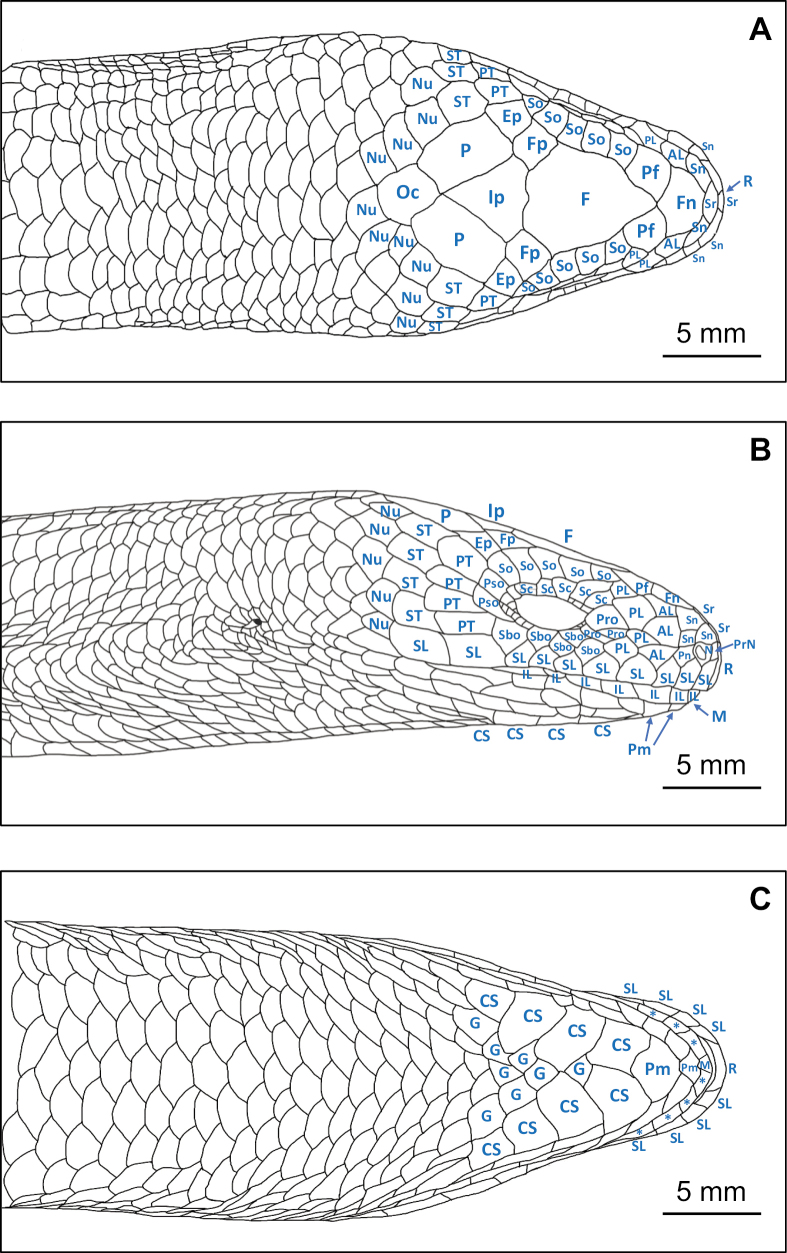
Head scalation of the neotype of *Dopasia formosensis* (NMNS 14488), illustrating the position, relative size, and terminology of head scales. Abbreviations: AL = anterior loreal, CS = chin shield, Ep = ectoparietal, F = frontal, Fn = frontonasal, Fp = frontoparietal, G = gular, IL or * = infralabial, Ip = interparietal, M = mental, N = nasal, Nu = nuchal, Oc = occipital scale, P = parietal, Pf = prefrontal, PL = posterior loreal, Pm = postmental, Pn = postnasal, Pro = preocular, Pso = postocular, PT = primary temporal, R = rostral, Sbo = subocular, Sc = superciliary, SL = supralabial, Sn = supranasal, So = supraocular, Sr = suprarostral, ST = secondary temporal. Illustrated by Chin-Chia Shen.

**75.3.3. Data ensuring recognition of the neotype**. External morphology is illustrated in Fig. 5 (dorsal and ventral views), with close-up images of the head provided in Fig. 6 (dorsal, lateral, and ventral views), and scalation terminology defined in Fig. 7. Presacral and caudal vertebral counts are documented via X-ray (Fig. 5C, Suppl. material 2). Morphometric and scalation data for the neotype and comparative series are summarized in Table 3 (see Suppl. material 1 for full measurements). Mitochondrial sequence data are deposited in GenBank under accession numbers PX229999, PX229448, and PX236186 (Table 1).

**75.3.4. Evidence that the original name-bearing type is lost, and steps taken to trace it**. Kyukichi Kishida (1930) described *Ophisaurus formosensis* based on a single specimen without a catalog number or stated repository. The specimen was collected by Harukichi Sato, a biology professor at the “High School of Taihoku” (now National Taiwan Normal University, NTNU). Although NTNU still preserves a portion of the vertebrate material from that period, no *Dopasia* specimens from that era are present; the type material is evidently no longer in Taiwan. At the time of publication, Kishida was affiliated with the Institute for Mammalogy and Ornithology, Imperial Agricultural Experiment Station, Nishigahara, Tokyo, which focused on avian and mammalian specimens. It was later incorporated into Japan’s National Agriculture and Food Research Organization, which no longer maintains natural history collections as a core function. Specimens of amphibians and reptiles were later transferred to the National Museum of Nature and Science (Tokyo). However, based on direct communication with curator and researcher Dr. Natsuhiko Yoshikawa from the Department of Zoology at that museum, we confirmed that the type specimen is no longer present in their collection. The only specimen remotely matching the relevant criteria is a *Dopasia harti* (NSMT-H2529), collected by Masao Uenishi on 2 June 1938 from “Nanpo-zan Mountain in the Formosan forest for the practice of Kyoto Imperial University”. Located in southern Taiwan, it clearly differs from the collection date and locality reported for the original type specimen from northern Taiwan. This specimen is therefore not the holotype of *D. formosensis*, and we conclude that the name-bearing type has been lost.

**75.3.5. Consistency with the former name-bearing type**. Lin et al. (2003) and our genetic and phylogenetic analyses both indicate that only a single *Dopasia* species occurs in Taiwan and that the presence or absence of bluish markings is non-diagnostic (Fig. 2). We therefore sought a neotype from near the original type locality.

**Figure 8.**
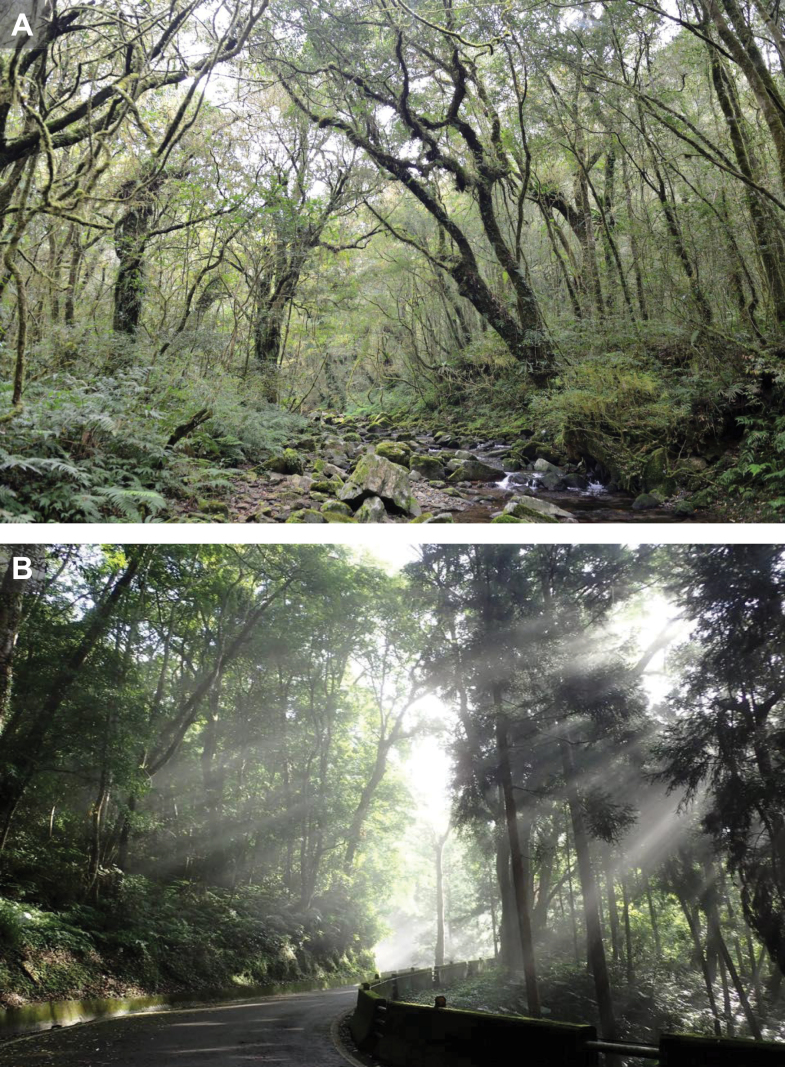
**A**. Landscape near the type locality of *Dopasia formosensis* (Kishida, 1930) in Hinokiyama, currently known as a part of the Fuba Cross-ridge Trail; **B**. Landscape of the collection site of the neotype and two topotypes (NMNS 14488, 14489, 14490) near Mingchi, Yilan, Taiwan. Photographed by Chung-Wei You and Kai-Xiang Chang.

**75.3.6. Provenance relative to the type locality**. Kishida’s type locality “Hinokiyama (= Hinoki Mountain), Taihoku-shiu (= Taipei Prefecture)” (1,400 m a.s.l.) corresponds to the modern Fuba Cross-ridge Trail (Fig. 8A). Due to its proximity to the Hapen Nature Reserve, the area has remained largely undeveloped for nearly a century, making direct sampling at the original locality difficult. The neotype locality (Mingchi; 24.6505°N, 121.4675°E, 1,133 m a.s.l.; Fig. 8B) lies within the same mid-elevation, humid broadleaf-forest belt, forming a continuous habitat block with Hinokiyama and situated ~ 8 km to the south of the historical site. Populations in Mingchi are continuous with and conspecific to all the samples from northern Taiwan, as supported by morphology and multilocus phylogenetic analyses (Fig. 2).

**75.3.7. Deposition**. The neotype (NMNS 14488) will remain the property of the National Museum of Natural Science (NMNS), Taichung, Taiwan, a recognized scientific institution that maintains research collections with appropriate facilities for preserving name-bearing types and provides access to qualified researchers.

**Neotype**. NMNS 14488 (Figs 5, 6, 7), adult male in a good state of preservation, collected by Wen-Hsian Chang and Si-Min Lin in May 2003 from Mingchi, Yilan County, Taiwan (24.6505°N, 121.4675°E) at an elevation of 1,133 m a.s.l. (Fig. 8B). This habitat represents a mid-elevation montane forest margin, where secondary growth and undisturbed natural forest coexist along a narrow mountain road. Despite the presence of anthropogenic structures, the site remains ecologically intact, with dense canopy cover, high humidity, and minimal disturbance to the forest floor. Early morning mist and frequent orographic cloud cover maintain moist microhabitats throughout much of the year.

**Topotypes**. Morphological analyses (*n* = 13): Six males, four females, and three juveniles (Table 1). NMNS 14489 (♂) and 14490 (♂) from Mingchi, Yilan County; NMNS 14491 (♀) from Yangmingshan, Taipei City; NMNS 14492 (♂) from Guanwu, Miaoli County; NMNS 14493 (♂) from Yangmingshan, Taipei City; NMNS (♀) 14494 from Siji, Yilan County; NMNS 14495 (♂) from Fushan, New Taipei City; NMNS 14496 (♀) from Chatianshan, New Taipei City; NMNS 14497 (♂) from Yangmingshan, Taipei City; NMNS 14498 (♀), locality unknown; NMNS 14500 and 14501 (both unsexed juveniles) from Yangmingshan, Taipei City; NMNS 14505 (juvenile ♀) from Northern Cross-island Highway. Most the above specimens were collected by Wen-Hsien Chang, Yu-Jen Liang, and Si-Min Lin during 1998 to 2005.

In addition to above-mentioned materials, we also included the following in our genetic analyses: NMNS 14504 (Xuejian); TBRI RN0070 (Zhongpu); TBRI RN0157 (Yangmingshan); TBRI RN2518 (Dahsuehshan); TBRI RN0588 (Siling); TBRI RN0904 (Tangzhi); TBRI RN1812 (Baling); TBRI RN1700 (Dahanshan); TBRI RN2851 (Siling) (Table 1).

**Diagnosis**. *Dopasia formosensis* is a medium-sized, limbless anguid lizard. Snout-vent length of adult males ranging between 175–230 mm (mean ± SD = 200.7 ± 22.4 mm); of adult females between 171–231 mm (mean ± SD = 201.3 ± 30.0 mm) (Table 3, Suppl. material 1). *Dopasia formosensis* has the following combination of morphological characters: intact tail length relatively long, ranging 1.74–1.95 (1.83 ± 0.09) of SVL and ranging 0.63–0.66 (0.65 ± 0.01) total length (*n* = 6, specimens with intact tails only). The body cross-section is approximately quadrangular, with slight dorsoventral compression; body width always wider than body height, BW1/BH1 ranging 1.00–1.32 at one-head length posterior of neck, BW2/BH2 1.01–1.22 at mid SVL, BW3/BH3 1.00–1.20 at one-head length anterior of cloaca, TW/TH 0.93–1.20 at one head-length posterior of cloaca (*n* = 14). Head relatively short compared to body length, HL/SVL 0.089–0.119; neck (could be defined as ear–fold distance) shorter than head, ErFD/SVL 0.065–0.103; head length longer than head width and head height, HW/HL 0.649–0.771, HH/HL 0.512–0.695 (*n* = 14 for the above measurements). External ear opening present but small, ear opening diameter (ErD) always smaller than nostril diameter (ND); eye subcircular to slightly oval in shape, moderate in size, EyS/HL 0.135–0.194; pupil round (Table 3).

Scales on head, neck, and anterior part of the body smooth; weakly keeled posterior to midbody, and strongly keeled posterior to cloaca. Dorsal scales longitudinal (DSL) 117–123 (mean ± SD = 119.3 ± 2.1); ventral scales longitudinal (VSL) 120–130 (124.9 ± 3.0); scales along lateral fold (SLF) 97–101 (99.2 ± 1.3); dorsal scales rows (DSR) 14 (57%) or 16 (29%), occasionally 18 (14%; among examined individuals, with the following percentages having the same definition); ventral scale rows (VSR) 10; caudal scale rows (CR) 22 (43%) or 24 (50%), occasionally 23 (7%). Supranasals present, separated by suprarostrals and frontonasal; prefrontals two, either separated by frontonasal and frontal or in contact with each other; frontal elongated, length/width 1.062–1.554, in contact with prefrontal anteriorly, with 1^st^ to 3^rd^ supraoculars laterally, with frontoparietals and interparietal posteriorly; frontoparietals two, separated by frontal and interparietal; interparietal large, diamond-shaped, in contact with frontal and frontoparietals anteriorly, with parietals laterally, with occipital scale posteriorly; parietals two, in contact posteriorly; enlarged occipital scale fan-shaped, could be distinguished by surrounding nuchals. Supraoculars generally five, occasionally four (7.1%); supralabials 11 (59%) or 10 (35%), occasionally 12 (6%); infralabials 10–13. Chin shields in four pairs, 1^st^ and 2^nd^ pairs in contact (Table 3, Suppl. material 1).

Number of vertebrae from the atlas to the remnants of the hind limb 54–56, mean ± SD = 54.8 ± 0.8 (*n* = 14). For individuals with intact tail, number of vertebrae (caudal vertebrae included) range between 146 and 153 (150.0 ± 2.9) (*n* = 5) (Table 3, Suppl. material 2).

#### Description of neotype

***Measurements of body*** (all in mm): total length (TotL) 605; snout–vent length (SVL) 221; tail length (TaL) 384; body width posterior of neck (BW1) 15.8; body height posterior of neck (BH1) 14.0; body width at mid SVL (BW2) 17.1; body height at mid SVL (BH2) 14.5; body width anterior of cloaca (BW3) 15.4; body height anterior of cloaca (BH3) 13.4; tail width (TW) 11.6; tail height (TH) 11.1.

***Measurements of head*** (all in mm; left/right if available): head length (HL) 21.9; head width (HW) 15.4; head height (HH) 13.1; nostril diameter (ND) 0.7/0.7; eye size (EyS) 3.4/4.0; ear opening diameter (ErD) 0.6/0.6; snout–eye distance (SEyD) 8.9/9.3; snout–ear distance (SErD) 25.4/25.1; snout–fold distance (SFdD) 41.9/41.0; nostril–eye distance (NEyD) 6.8/6.8; nostril–ear distance (NErD) 23.5/23.5; eye–ear distance (EyErD) 13.3/13.6; ear–fold distance (ErFD) 16.9/16.3; mouth length (MoL) 19.8/19.9; mandible length (MaL) 20.6/20.4; snout–frontal distance (SFtD) 5.7; frontal shield length (FL) 8.0; frontal shield width (FW) 5.7; rostrum width (RW) 2.8; nostril width (NW) 4.2; jaw width (JW) 14.9.

Adult male in a good state of preservation (Figs 5, 6, 7). Body elongated, SVL over ten times of HL (HL/SVL 0.099); tail longer than body (TaL/SVL 1.74), comprising of total length (TaL/TL 0.58). Head comparatively small (HL/SVL 0.15; TL/HL 15.6), elongate, notably longer than wide (HW/HL 0.70); head height less than head width (HH/HW 0.85; HH/HL 0.60). Neck (defined as ErFD) relatively short (ErFD/HL 0.77; ErFD/SVL 0.076). Snout rounded in profile in dorsal view; ear opening small, ear opening size smaller than nostril size (EaS/NS 0.83). Eyes medium sized (EyS/HL 0.17); eye–ear distance more than a half of head length (EyErD/HL 0.61).

***Scale counts***: Dorsal scales longitudinal (DSL) 123; ventral scales longitudinal (VSL) 126; scales along lateral fold (SLF) 100/99; dorsal scales rows (DSR) 14; ventral scale rows (VSR) 10; caudal scale rows (CR) 24; supraocular (So) 5/5; superciliary (Sc) 5/5; supralabial (SL) 10/10; and infralabial (IL) 11/10.

***Dorsal view of head scalation***: Rostral roughly trapezoidal, width equal to height, not visible from above and almost invisible in lateral view, in contact with 1^st^ supralabial laterally and suprarostral posteriorly; suprarostral two, flat hexagonal, separating rostral and frontonasal; supranasals present, three on each side, two located basally near the nostril and one dorsally, separating nostril from frontonasal; frontonasal broad, pentagonal in shape, in contact with 2^nd^ suprarostral anteriorly, supranasal and anterior loreal laterally, and prefrontal posteriorly; prefrontals paired, separated medially by frontonasal and frontal, slighted connected at the posteromedial corners, each in contact with frontonasal and anterior loreal anteriorly, posterior loreal laterally, 1^st^ supraocular and frontal posteriorly; frontal elongate hexagonal, distinctly longer than wide (FL/FW 1.40), in contact with frontonasal and prefrontals anteriorly, 1^st^ – 3^rd^ supraoculars laterally, frontoparietals and interparietal posteriorly; frontoparietals paired, roughly pentagonal, separated along the midline between frontal and interparietal, contacting frontal, 3^rd^ and 4^th^ supraoculars anteriorly, 5^th^ supraocular laterally, ectoparietal, parietal, and interparietal posteriorly; interparietal large, diamond-shaped, in contact with frontal and frontoparietals anteriorly, parietals laterally, occipital plate posteriorly; parietals paired, contacting each other posteriorly, in contact with interparietal, frontoparietal, and ectoparietal anteriorly, with secondary temporal and nuchals laterally, with nuchals and occipital plate posteriorly; occipital plate enlarged, fan-shaped, in contact with parietals anteriorly, with nuchals laterally and posteriorly (Figs 6A, 7A).

***Lateral view of head scalation***: Naris oval-shaped, nasals in contact with a small prenasal anteriorly, two supranasals dorsally, a small postnasal anteriorly, 1^st^ and 2^nd^ supralabials ventrally; loreals seven, aligned in two rows, three anterior and four posterior; anterior loreals three, aligned vertically, in contact with supranasals and postnasal anteriorly, with frontonasal and prefrontal dorsally, with posterior loreals posteriorly, with 3^rd^ and 4^th^ supralabials ventrally; posterior loreals four, aligned vertically, in contact with anterior loreals anteriorly, with prefrontal and supraocular dorsally, with supracilaries, preoculars, and subocular posteriorly, with 4^th^ and 5^th^ supralabials ventrally; preoculars three, the upper one much larger than the lower two; supraoculars five, approximately the same size, 1^st^ contacting prefrontal and frontal, 2^nd^ and 3^rd^ contacting frontal, 4^th^ contacting frontoparietal, 5^th^ contacting ectoparietal; supraciliaries six, 2^nd^ the largest; suboculars five, approximately the same size with supralabials, contacting 5^th^ to 9^th^ supralabials ventrally; postoculars two, in contact with supraocular dorsally, with primary temporal posteriorly, with 5^th^ subocular ventrally; primary temporals four, aligned vertically, in contact with supraocular, postoculars, and subocular anteriorly, with ectoparietal dorsally, with secondary temporals posteriorly, with 9^th^ supralabial ventrally; secondary temporals four, aligned vertically, in contact with primary temporals anteriorly, with parietal dorsally, with nuchals posteriorly, with 10^st^ supralabial ventrally; supralabials ten, 9^th^ and 10^th^ the largest, 6^rd^ to 8^th^ located below the eye; infralabials ten, 1^st^ invisible in lateral view; upper lip extends slightly over the lower lip, partially concealing infralabials 8 to 10 beneath its margin; two to three rows of rectangular scales between infralabials and chin shields, aligned parallel to the infralabials, similar in size and shape (Figs 6B, 7B).

***Ventral view of head scalation***: Mental scale subtriangular, in contact with 1^st^ infralabial laterally, with 1^st^ postmental posteriorly; postmentals two, 1^st^ postmental pentagonal, in contact with mental and 1^st^ infralabial anteriorly, with the rectangular scales laterally, with 2^nd^ postmental posteriorly; 2^nd^ postmental enlarged, diamond-shaped, in contact with 1^st^ postmental and the rectangular scales anteriorly, 1^st^ pair of chin shields posteriorly; chin shields in four pairs, 1^st^ pair in broad contact medially, forming a distinct suture, contacting 2^nd^ postmental anteriorly, the rectangular scales laterally, 2^nd^ pair of chin shields and 1^st^ gular posteriorly; 2^nd^ chin shields separated anteriorly by the diamond-shaped 1^st^ gular, connected posteriorly; 3^rd^ pair of chin shields separated medially by three gulars; 4^th^ pair by five gulars. The upper lip slightly overlaps the lower lip; the rostral, the 1^st^ to 4^th^ supralabials, and the 1^st^ to 4^th^ infralabials are visible in ventral view (Figs 6C, 7C).

***Number of vertebrae***: Presacral vertebrae (VPS) 56; caudal vertebrae (VC) 97; total vertebrae (VT) 153 (Fig. 5C).

**Figure 9.**
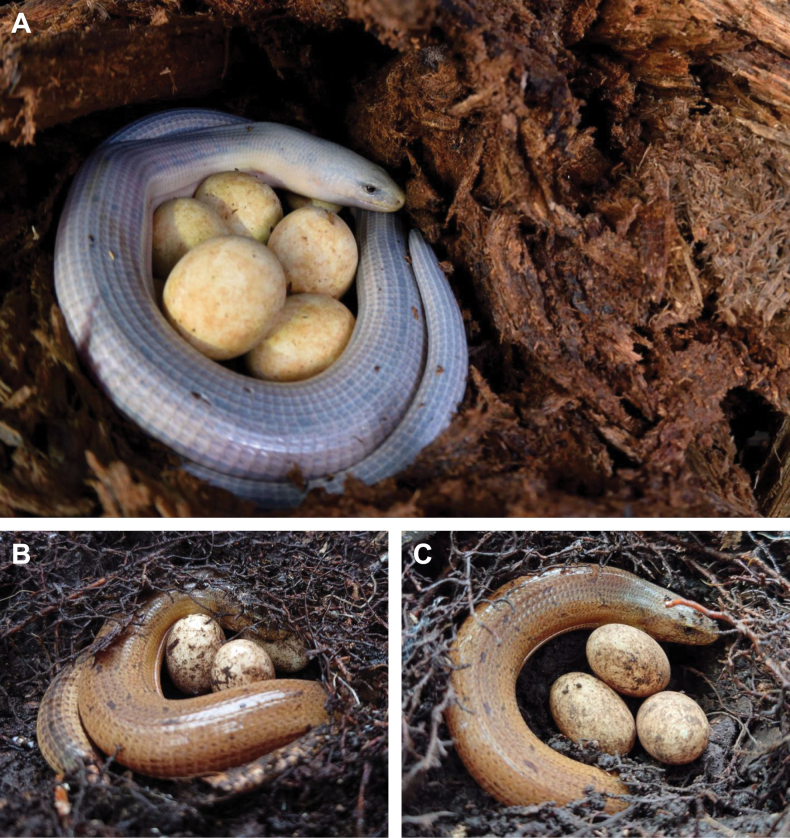
**A**. Egg guarding by a female *Dopasia formosensis* in the wild, first photographed by Dash Huang in 2015 and reported by Jablonski and Lawson (2020). The conspicuous grayish-white coloration documented in this individual is here interpreted as a pre-ecdysis condition and considered indicative of prolonged nest attendance. Photograph by Dash Huang, first published in Jablonski and Lawson (2020: Fig. 1), and reproduced with permission of the photographer; **B, C**. In a subsequent case documented in 2023, the same female remained in close contact with her eggs across multiple observation events, indicating continued egg attendance despite moderate disturbance (photographs by Weizun Wang). Both cases were observed in Pingtung County, southern Taiwan.

**Coloration in life**. Dorsal coloration of head, nape, trunk, and tail uniform, typically reddish-brown, yellowish-brown, ochre, or tawny (Fig. 4A, B). Loreal and orbital regions slightly darker; ventral surface marginally paler, but without a distinct boundary separating it from the flanks and dorsum. In some individuals, a pair of narrow and faint dark longitudinal lines is present along the junction between the lateral and dorsal body surfaces. Adult males exhibit 12–18 irregular bluish transverse bands on the dorsum, which tend to increase in number and extent with sexual maturity. Females usually greyish-brown, lack these blue markings, have either a completely uniform dorsum or bears only faint, narrow, black transverse lines (Fig. 9). Juveniles lack dorsal markings and exhibit a pale brown dorsum. Lateral and ventral surfaces deep black, with the black pigmentation extending continuously from the narial region across the loreal and orbital regions, postorbital area, and temporal region, continuing posteriorly along the flanks and tail (Fig. 4C). Individuals approaching ecdysis exhibit a paler coloration and may even turn entirely grayish-white several days prior to shedding, as demonstrated in the photograph from Jablonski and Lawson (2020).

**Coloration in preservative**. The overall color pattern remains recognizable after preservation (Fig. 5). In males, the original reddish- or yellowish-brown dorsal coloration typically fades to greyish-brown, but the bluish transverse markings remain clearly visible. In females, coloration shows minimal change, and the faint longitudinal dorsolateral stripes and transverse dark lines are generally retained.

**Variation**. The number of dorsal scale rows was historically considered a diagnostic character for distinguishing *D. harti* (16 rows) from *D. formosensis* (14 rows). However, both Lin et al. (2003) and the present study found that the number of dorsal scale rows exhibits some degree of variation and cannot reliably be used as a diagnostic character. In particular, in specimens that were relatively emaciated at the time of preservation, the lateral skin folds may cover one or even two dorsal scale rows, leading to undercounts during examination. Moreover, the dorsal scales located near the base of the lateral folds tend to become markedly smaller, and whether these smaller scales should be included in the dorsal scale row count can also introduce inconsistency in scoring.

In this study, we carefully re-examined all well-preserved specimens and excluded the influence of small marginal scales by counting only the large, regular dorsal scale rows. As a result, the number of dorsal scale rows in *D. formosensis* was found to range from 14 to 18, with 14 rows observed in 57% of individuals (*n* = 8), 16 rows in 29% (*n* = 4), and 18 rows in 14% (*n* = 2). These findings suggest that although variation in dorsal scale row number does occur within the species, *D. formosensis* does exhibit a higher tendency toward having 14 dorsal scale rows when smaller marginal scales are excluded from the count.

Additional morphological features that may be relevant for discussion include the number of caudal scale rows, which ranges from 22 to 24. Among these, 24 (50%) and 22 (43%) are the most frequently observed counts, while 23 is relatively rare (7%). Supraoculars typically five, although in a few individuals (*n* = 2; 14%) only four were observed on one side. Supraciliaries usually five, but sometimes four (29%) or six (7%). Supralabials usually 11 (63%), followed by 10 (33%), with a rare case of 12 (4%). The number of infralabials varies from 10 to 13. However, we note that due to the slight overlap of the upper lip over the lower lip in *Dopasia*, the most basal infralabials may sometimes be obscured and not externally visible, making accurate counts difficult.

**Etymology**. The specific epithet *formosensis* is an adjective derived from Formosa, the historical western name for Taiwan. The name Formosa originates from the Portuguese expression Ilha Formosa (“beautiful island”), reportedly given by Portuguese sailors passing the island in the mid-16^th^ century. The epithet thus means “from Formosa,” in reference to the island of Taiwan, the type locality of the species.

**Distribution and habitat preference**. *Dopasia formosensis* is known from montane areas at low to mid elevations, with currently confirmed records ranging from approximately 500 to 2000 m above sea level (Shang et al. 2009). Although exhibits a broad geographic distribution across montane regions of Taiwan, it remains exceedingly rare throughout its range. Field encounters are infrequent, suggesting that the species may occur at low population densities or exhibit high microhabitat preferences.

*Dopasia formosensis* is presumed to be strongly associated with specific habitat conditions, particularly cool, humid montane environments with intact native vegetation and a well-developed organic layer on the forest floor (Fig. 8A). Suitable habitats are expected to consist of pristine, mid-elevation broadleaf forests characterized by high canopy closure and a multi-layered vegetation structure. The microclimate remains consistently moist due to frequent precipitation and dense canopy cover, facilitating the accumulation of a thick, moisture-retentive leaf litter layer. The forest floor typically supports abundant decomposing plant material and a deep humus horizon, enhancing soil fertility and sustaining invertebrate populations. Mature trees are densely distributed and frequently covered with epiphytic mosses and lichens, indicative of prolonged humidity. The substrate is generally dominated by mossy rocks, ferns, and accumulated leaf litter, offering ideal shelter and oviposition sites for fossorial or cryptozoic reptiles.

Well-documented localities of *Dopasia formosensis* include Yangmingshan (particularly along the Bailaka Road), sites along the Northern Cross-island Highway (e.g., Baling, Siling, Mingchi, Qilan), as well as Nanao, Dahsuehshan, Xitou, the Alishan Highway, Tengzhi, and Dahanshan. Among these, Yangmingshan and the Northern Cross-island Highway in northern Taiwan represent the areas with the most frequent records. However, it remains unclear whether this reflects genuinely larger population sizes or simply greater survey effort and reporting by local enthusiasts. Observations have also been reported from eastern Taiwan, although this area remains relatively under-surveyed.

**Natural history notes**. The species is highly secretive and appears to spend most of its time within the moist understory of intact natural forests. Consequently, its ecology can only be inferred from fragmentary observations. Roadkill and field sightings peak from April to June, suggesting that this is the principal period of activity. Remarkable higher detection rate in males rather than females indicates a higher male mobility during the breeding season. During this season, males exhibit pronounced aggressiveness (Lawson et al. 2026; SML, pers. obs.), including a ritualized threat display in which the anterior body is elevated, the neck is slightly laterally flattened, the head is pressed downward to an extreme angle, with the mouth slightly agape. This posture is maintained motionless for several seconds before vigorous biting ensues. The record by Lawson et al. (2026) further indicates that serious fighting between males can result in tail loss.

Oviparous, clutch size might be three to six. Eggs short-ellipsoid, leathery, and relatively large, with egg diameters nearly equal to the body width of the mother. Females exhibit pronounced egg-guarding behavior by coiling around the clutch, a behavior that has been repeatedly observed and photographed. Jablonski and Lawson (2020) reported the first case of an egg-guarding female exhibiting a conspicuous grayish-white coloration associated with pre-ecdysis, suggesting prolonged attendance at the nest (Fig. 9A). Similar observations have since been documented (Fig. 9B, C). In some cases, egg-guarding females were approached on multiple occasions by different photographers; nevertheless, the females remained at the nest site and continued attending the clutch for extended periods.

Under stress, individuals undergo caudal autotomy. The wound heals, but the tail does not regenerate or elongate. In the wild, approximately 60% of individuals show signs of previous tail loss, with only ~ 40% retaining an intact tail.

Limited husbandry observations in the early years (late 1990s to early 2000s) suggested a strong dietary specialization on earthworms, as captive individuals consistently rejected most alternative prey items. Compared with many other lizards, individuals exhibit a low feeding frequency, consistent with relatively low metabolic demands, and can be maintained on small amounts of food while retaining excellent body condition. The breadth of the diet in the wild remains poorly known and warrants further study.

**Conservation status**. Although broadly distributed in Taiwan, *Dopasia formosensis* is consistently rare throughout its range. No robust data are currently available to assess long-term trends in population size or trajectory. Due to its apparent specialization for specific microhabitat conditions, such as cool, moist climates, intact forest canopy, and a well-developed humus layer, the conservation of native forests is likely essential for the persistence of this species. According to The Red List of Terrestrial Reptiles of Taiwan, the species was assessed as Data Deficient (DD) in the 2017 version (Chen et al. 2017) and updated to Least Concern (LC) in 2024 (Hsu et al. 2024). Nevertheless, in view of its rarity and to prevent unregulated collection, *Dopasia formosensis* is currently listed under Category III (other species requiring conservation) of Taiwan’s Wildlife Conservation Act.

## Additional information

### Use of AI

No use of AI was reported.

### Author ORCIDs

Si-Min Lin https://orcid.org/0000-0001-7080-706X

Chin-Chia Shen https://orcid.org/0009-0006-0203-9007

Te-En Lin https://orcid.org/0000-0002-0920-4937

### Data availability

All of the data that support the findings of this study are available in the main text or Supplementary Information.

## Supplementary materials

Supplementary material 1 Measurements and scale counts Data type xlsx Explanation note This file contains all measurement and count data for *Dopasia formosensis*, including the neotype, neoparatypes, and other specimens examined. https://binary.pensoft.net/file/1544394This dataset is made available under the Open Database License (http://opendatacommons.org/licenses/odbl/1.0/). The Open Database License (ODbL) is a license agreement intended to allow users to freely share, modify, and use this Dataset while maintaining this same freedom for others, provided that the original source and author(s) are credited.Author: Si-Min Lin

Supplementary material 2 Supplementary image Data type pdf Explanation note This file contains X-ray photographs of all well-preserved specimens of *Dopasia formosensis*, including the methods used to determine presacral, caudal, and total vertebrae counts. https://binary.pensoft.net/file/1544395This dataset is made available under the Open Database License (http://opendatacommons.org/licenses/odbl/1.0/). The Open Database License (ODbL) is a license agreement intended to allow users to freely share, modify, and use this Dataset while maintaining this same freedom for others, provided that the original source and author(s) are credited.Author: Si-Min Lin

## References

[B1] Boulenger GA (1899) On a collection of reptiles and batrachians made by Mr. J. D. La Touche in N. W. Fokien, China. Proceedings of the Zoological Society of London 11: 159–172. 10.1111/j.1469-7998.1899.tb06855.x

[B2] Brygoo ER (1987) Les Ophisaurus (Sauria, Anguidae) d’Asie orientale. Bulletin du Muséum national d’Histoire naturelle (Paris), Série 4 9: 727–752. 10.5962/p.287541

[B3] Cai B, Guo X, Song Z, Chen D (2020) The complete mitochondrial genome of the Hainan Glass Lizard (*Dopasia hainanensis*) determined by next-generation sequencing. Mitochondrial DNA Part B 5: 246–247. 10.1080/23802359.2019.1700194 PMC7748677 33366506

[B4] Castoe TA, Jiang ZJ, Gu W, Wang ZO, Pollock DD (2008) Adaptive evolution and functional redesign of core metabolic proteins in snakes. PLoS ONE 3: e2201. 10.1371/journal.pone.0002201 PMC2376058 18493604

[B5] Chen JTF, Yu MJ (1984) A synopsis of the vertebrates of Taiwan, revised and enlarged edition (in 3 Vols), Vol. III. Commercial Press, Taipei, Taiwan, 633 pp.

[B6] Chen YL, Lin TE, Lin RS, Yang CH (2017) The Red Lists of Terrestrial Reptiles of Taiwan, 2017. Endemic Species Research Institute, Committee of Agriculture, Nantou, Taiwan, 35 pp. https://www.tbri.gov.tw/redirect_file.php?theme=web_structure&id=4839

[B7] Conrad JL, Norell MA (2008) The braincases of two glyptosaurines (Anguidae, Squamata) and anguid phylogeny. American Museum Novitates 3613: 1–24. 10.1206/586.1

[B8] Conrad JL, Ast JC, Montanari S, Norell, MA (2010) A combined evidence phylogenetic analysis of Anguimorpha (Reptilia: Squamata). Cladistics 26: 1–48. 10.1111/j.1096-0031.2010.00330.x 34875778

[B9] Gvoždík V, Jandzik D, Lymberakis P, Jablonski D, Moravec J (2010) Slow worm, *Anguis fragilis* (Reptilia: Anguidae) as a species complex: Genetic structure reveals deep divergences. Molecular Phylogenetics and Evolution 55: 460–472. 10.1016/j.ympev.2010.01.007 20079858

[B10] Gvoždík V, Benkovský N, Crottini A, Bellati A, Moravec J, Romano A, Sacchi R, Jandzik D (2013) An ancient lineage of slow worms, genus *Anguis* (Squamata: Anguidae), survived in the Italian Peninsula. Molecular Phylogenetics and Evolution 69: 1077–1092. 10.1016/j.ympev.2013.05.004 23702464

[B11] Hoang DT, Chernomor O, Haeseler AV, Minh BQ, Vinh LS (2018) UFBoot2: Improving the ultrafast bootstrap approximation. Molecular Biology and Evolution 35: 518–522. 10.1093/molbev/msx281 PMC5850222 29077904

[B12] Hsu FH, Lin SM, Yang CK, Lin TE (2024) The Red List of Terrestrial Reptiles of Taiwan, 2024. Taiwan Biodiversity Research Institute, Ministry of Agriculture, Nantou, Taiwan; Forestry and Nature Conservation Agency, Ministry of Agriculture, Taipei, Taiwan, 35 pp. https://www.tbri.gov.tw/redirect_file.php?theme=web_structure&id=4354

[B13] Jablonski D, Lawson M (2020) Remarks on coloration and shedding in a Hart’s glass lizard, *Dopasia harti* (Anguidae). Repiles and Amphibians 27: 452–453. 10.17161/randa.v27i3.14945

[B14] Jablonski D, Lawson M, Boyce AJ, Molls C, Das I (2020) An assessment of vouchered records and field observations of the rare anguid, *Dopasia buettikoferi* (Lidth de Jeude, 1905) in Borneo. Herpetozoa 33: 59–65. 10.3897/herpetozoa.33.e51089

[B15] Kalyaanamoorthy S, Minh BQ, Wong TKF, Haeseler AV, Jermiin LS (2017) ModelFinder: Fast model selection for accurate phylogenetic estimates. Nature Methods 14: 587–589. 10.1038/nmeth.4285 PMC5453245 28481363

[B16] Kishida K (1930) Notes on a Formosan lizard of the family Anguidae. Lansania 2: 124–128.

[B17] Kurita K, Nakamura Y, Okamoto T, Lin SM, Hikida T (2017) Taxonomic reassessment of two subspecies of Chinese skink in Taiwan based on morphological and molecular investigations (Squamata, Scincidae). ZooKeys 687: 131–148. 10.3897/zookeys.687.12742 PMC5672577 29114169

[B18] Lavin BR, Girman DJ (2019) Phylogenetic relationships and divergence dating in the Glass Lizards (Anguinae). Molecular Phylogenetics and Evolution 133: 128–140. 10.1016/j.ympev.2018.12.022 30584918

[B19] Lawson M, Su YH, Lin SM, Jablonski D (2026) First detailed description of male-male combat in the genus *Dopasia* Gray, 1875 (Reptilia: Anguidae). ACTA Ethologica 29: 3. 10.1007/s10211-025-00475-x

[B20] Li M, Wang Y, Wang L, Wang J, Cai B (2024) Taxonomic status of *Dopasia hainanensis*. Chinese Journal of Zoology 59: 233–244. 10.13859/j.cjz.202423062

[B21] Lin JY, Cheng HY (1990) Lizards of Taiwan. Taiwan Provincial Museum, Taipei, Taiwan, 176 pp.

[B22] Lin SM, Chang WS, Chen SL, Shang G, Lue KY (2003) Taxonomic status of the legless lizard *Ophiosaurus* (Squamata: Anguidae) in Taiwan: Molecular data, morphology, and literature review. Zoological Studies 42: 411–419. https://www.academia.edu/download/80392350/411.pdf

[B23] Lin TH, Shen ZY, Chou MH, Sun PW, Shan CC, Huang JP, Lin SM (2025) Allopatric speciation and interspecific gene flow driven by niche conservatism of *Diploderma* tree lizards in Taiwan. Molecular Ecology 34: e17718. 10.1111/mec.17718 40052357

[B24] Liu-Yu MC (1970) Studies on Taiwan lizards. Biological Bulletin of National Taiwan Normal University 5: 51–93.

[B25] Lue KY (1990) The manuals of wildlife resources inventory in Taiwan (2): The amphibians and reptiles of Taiwan. Council of Agriculture, Executive Yuan, Taipei, Taiwan, 123 pp.

[B26] Lue KY, Lin SM (2008) Two new cryptic species of *Takydromus* (Squamata: Lacertidae) from Taiwan. Herpetologica 64: 379–395. 10.1655/07-030.1

[B27] Lue KY, Chen SH, Chen YS, Chen SL (1987) Reptiles of Taiwan - lizards. Taiwan Provincial Department of Education, Taipei, Taiwan, 116 pp.

[B28] Lue KY, Tu MC, Shang G (1999) A field guide to the amphibians and reptiles of Taiwan. Society for Wildlife and Nature, Taipei, Taiwan, 343 pp.

[B29] Lue KY, Tu MC, Shang G (2002) A field guide to the amphibians and reptiles of Taiwan (2^nd^ edn.). Society for Wildlife and Nature, Taipei, Taiwan, 347 pp.

[B30] Minh BQ, Schmidt HA, Chernomor O, Schrempf D, Woodhams MD, Von Haeseler A, Lanfear R (2020) IQ-TREE 2: new models and efficient methods for phylogenetic inference in the genomic era. Molecular Biology and Evolution 37: 1530–1534. 10.1093/molbev/msaa015 PMC7182206 32011700

[B31] Nguyen TQ, Boehme W, Nguyen TT, Le QK, Pahl KR, Haus T, Ziegler T (2011) Review of the genus *Dopasia* Gray, 1853 (Squamata: Anguidae) in the Indochina subregion. Zootaxa 2894: 58–68. 10.11646/zootaxa.2894.1.4

[B32] Pan HC, Liu L, Li P, Li XF, Liu ZL (2015) The complete mitochondrial genome of Chinese glass lizard *Ophisaurus harti* (Squamata: Anguidae). Mitochondrial DNA 26: 2808–281. 10.3109/19401736.2013.825775 24020998

[B33] Papenfuss TJ, Parham JF (2013) Four new species of California legless lizards (*Anniella*). Breviora 536: 1–17. 10.3099/MCZ10.1

[B34] Posada D (2008) jModelTest: phylogenetic model averaging. Molecular Biology and Evolution 25: 1253–1256. 10.1093/molbev/msn083 18397919

[B35] Rao D [Ed.] (2024) Lizards of China. The Straits Publishing & Distributing Group, Fuzhou, China, 455 pp.

[B36] Ronquist F, Teslenko M, van der Mark P, Ayres DL, Darling A, Höhna S, Larget B, Liu L, Suchard MA, Huelsenbeck JP (2012) MrBayes 3.2: Efficient Bayesian phylogenetic inference and model choice across a large model space. Systematic Biology 61: 539–542. 10.1093/sysbio/sys029 PMC3329765 22357727

[B37] Shang G (2001) A Field Guide to Lizards in Taiwan. Bigtrees Press, Taipei, Taiwan, 173 pp.

[B38] Shang G (2007) Natural Portraits of Herpetofauna of Shei-Pa National Park. Shei-Pa National Park, Miaoli, Taiwan, 197 pp.

[B39] Shang G (2008) A Field Guide to Lizards in Taiwan. Commonwealth Publishing Group, Taipei, Taiwan, 173 pp.

[B40] Shang G, Li PH, Yang YJ (2009) A field guide to amphibians and reptiles in Taiwan. Owl Publishing, Taipei, Taiwan, 336 pp.

[B41] Stejneger L (1910) The batrachians and reptiles of Formosa. Proceedings of the United States National Museum 38: 91–114. 10.5479/si.00963801.1731.91

[B42] Stejneger L (1919) The“glass-snake”of Formosa. Proceedings of the Biological Society of Washington 32: 142. http://biographicalmemoirs.org/pdfs/stejneger-leonhard.pdf

[B43] Tamura K, Stecher G, Kumar S (2021) MEGA11: molecular evolutionary genetics analysis version 11. Molecular Biology and Evolution 38: 3022–3027. 10.1093/molbev/msab120 PMC8233496 33892491

[B44] Thanou, E, Kypraios-Skrekas V, Kornilios P, Giokas S (2021) Ecomorphological divergence and lack of gene flow in two sympatric Balkan slow worms (Squamata: Anguidae). Biological Journal of the Linnean Society 134: 443–460. 10.1093/biolinnean/blab074

[B45] Thompson JD, Higgins DG, Gibson TJ (1994) CLUSTAL W: improving the sensitivity of progressive multiple sequence alignment through sequence weighting, position-specific gap penalties and weight matrix choice. Nucleic Acids Research 22: 4673–4680. 10.1093/nar/22.22.4673 PMC308517 7984417

[B46] Tseng SP, Wang CJ, Li SH, Lin SM (2015) Within-island speciation with an exceptional case of distinct separation between two sibling lizard species divided by a narrow stream. Molecular Phylogenetics and Evolution 90: 164–175. 10.1016/j.ympev.2015.04.022 25982689

[B47] Van Denburgh J (1909) New and previously unrecorded species of reptiles and amphibians from the island of Formosa. Proceedings of the California Academy of Sciences, Fourth Series 3: 49–56.

[B48] Wang CS, Liang YS (1976) Notes on the reptiles found from upstream area between the rivers, Tatu Chi and Choshui Chi. I. Turtles and lizards. Life Science (Zoological Department, National Taiwan University) 7: 25–41.

[B49] Wang YT, Lin TH, Tseng HY, Poyarkov NA, Lin SM (2025) A new skink of the genus *Sphenomorphus* Fitzinger, 1843 from mid-elevation cloud forest of Taiwan. Herpetologica 81: 183–197. 10.1655/Herpetologica-D-24-00006

[B50] Yang RS (1983) A new species of the genus *Ophisaurus* from Hainan Island. Acta Herpetologica Sinica 2: 67–69. http://biodiversitylibrary.org/page/46027344

[B51] Zhan L, Chen Y, He J, Guo Z, Wu L, Storey KB, Zhang J, Yu D (2024) The phylogenetic relationships of major lizard families using mitochondrial genomes and selection pressure analyses in anguimorpha. International Journal of Molecular Sciences 25: 8464. 10.3390/ijms25158464 PMC11312734 39126033

