# Supplementary material for: Redescription and neotype designation of Dopasia formosensis (Kishida, 1930) (Squamata, Anguidae) from Taiwan
Source: Zookeys. 2026 Feb 19;1270:69–98. doi: 10.3897/zookeys.1270.173752 (PMC12946829; doi:10.3897/zookeys.1270.173752)
Supplement: Supplementary material 2 — Supplementary image [file zookeys-1270-069_article-173752__-s002.pdf]

NMNS 14488

Neotype, adult male, intact tail

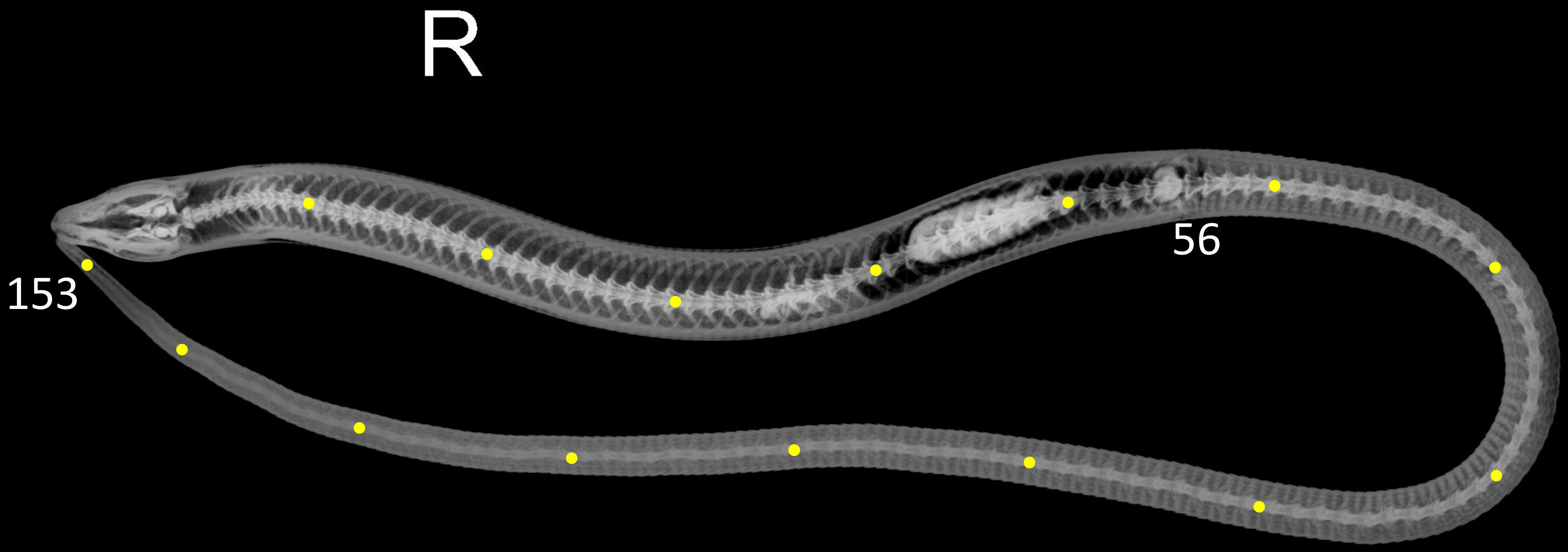

NMNS 14489

Paraneotype, adult male

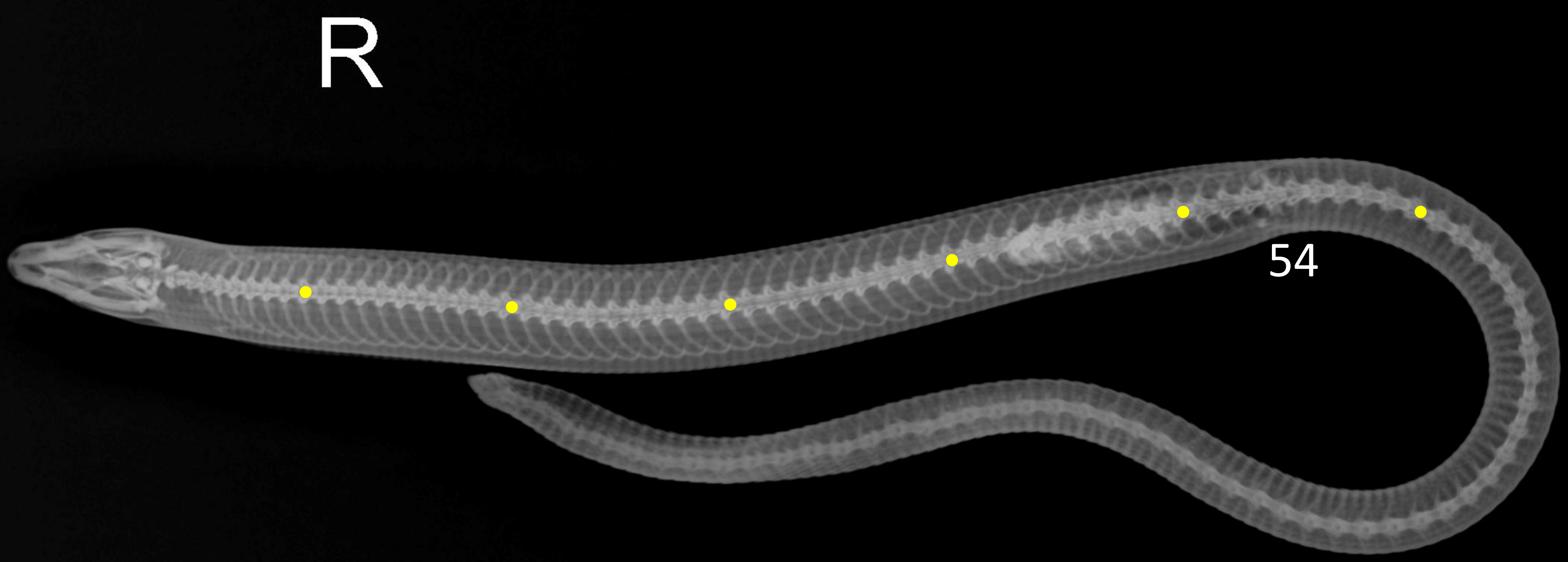

NMNS 14490
Paraneotype, adult male

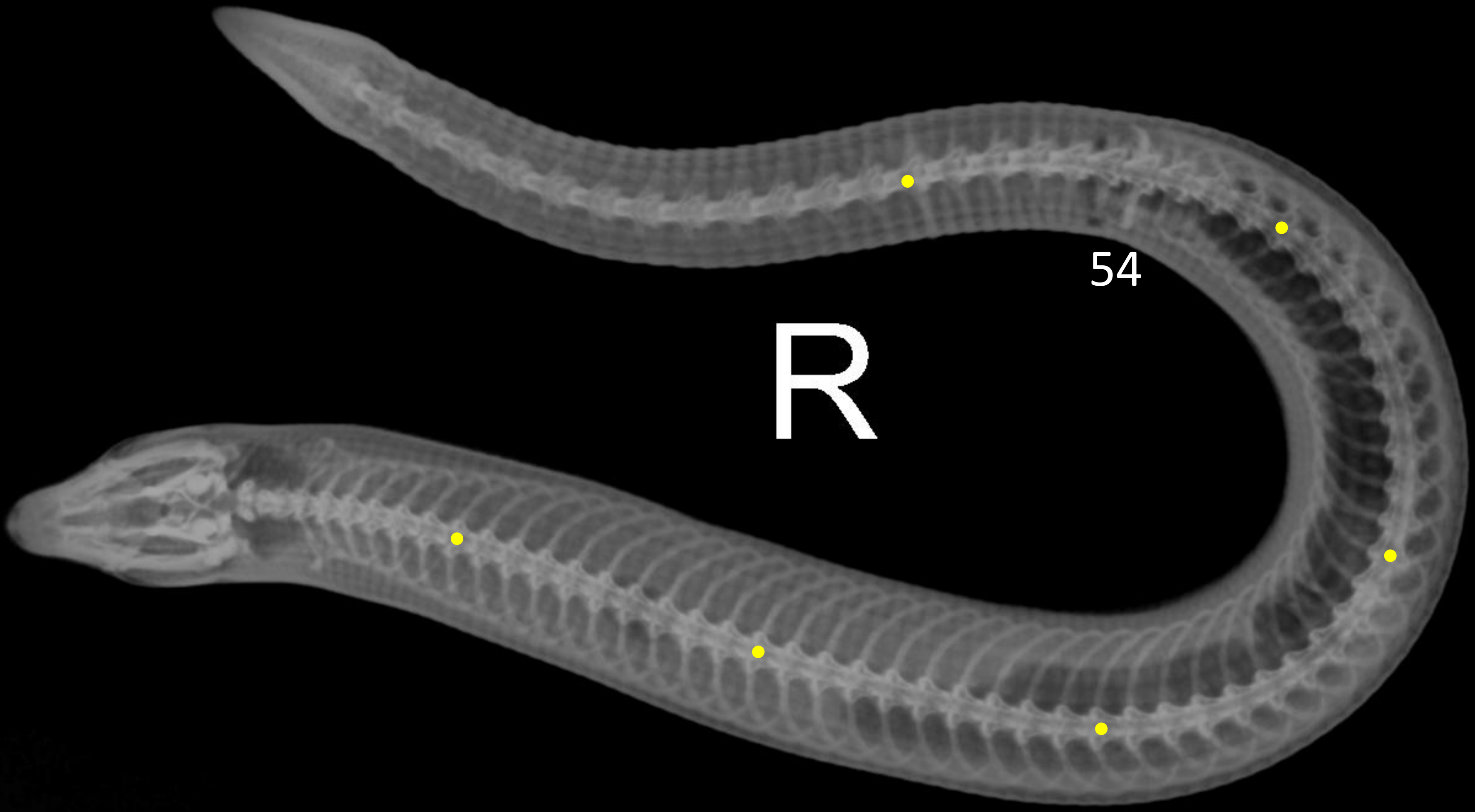

NMNS 14491
Paraneotype, adult female

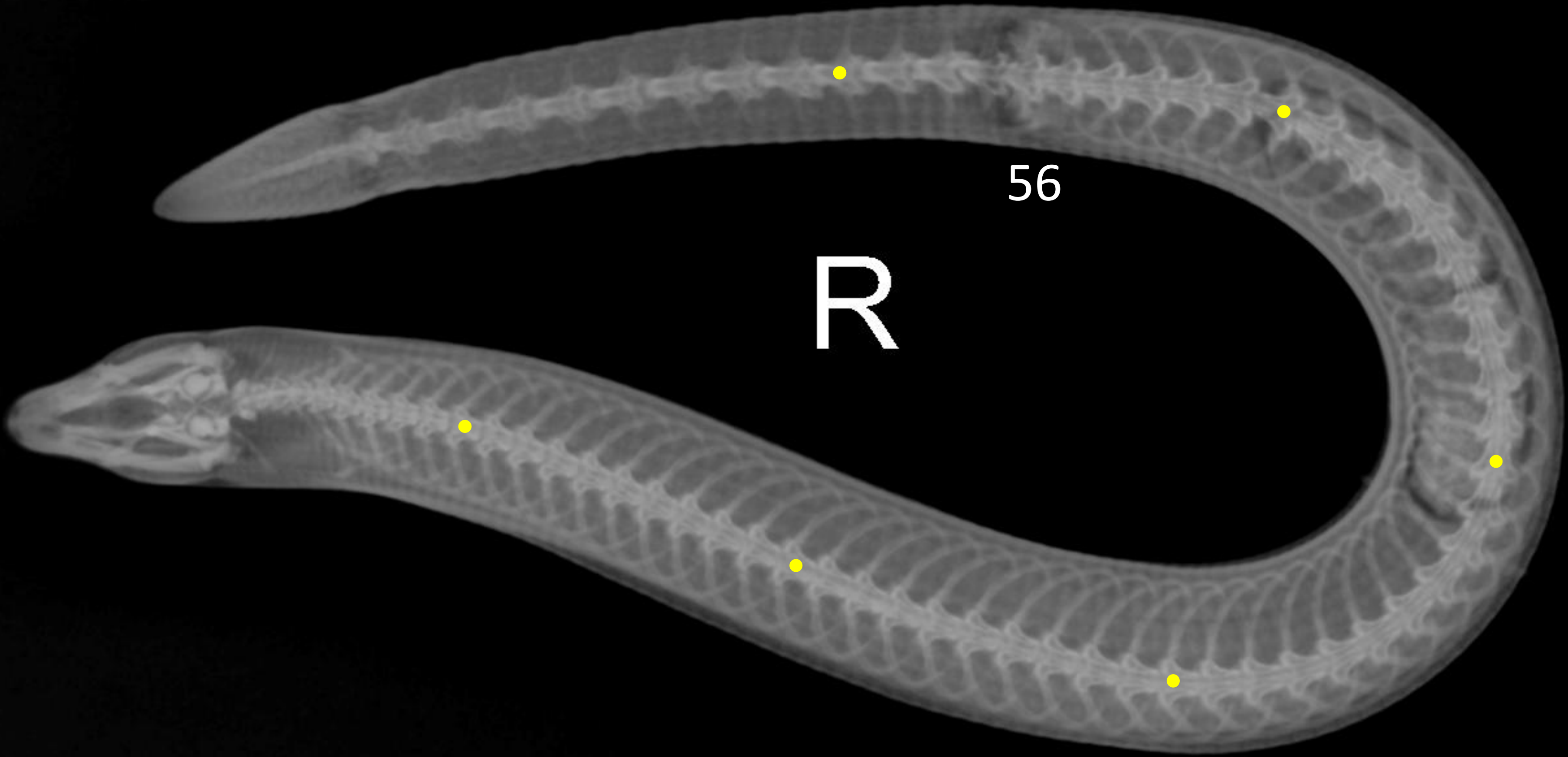

NMNS 14492

Paraneotype, adult male, intact tail*

* A short tail segment, equal to the underlying fragment, has been used for DNA extraction.

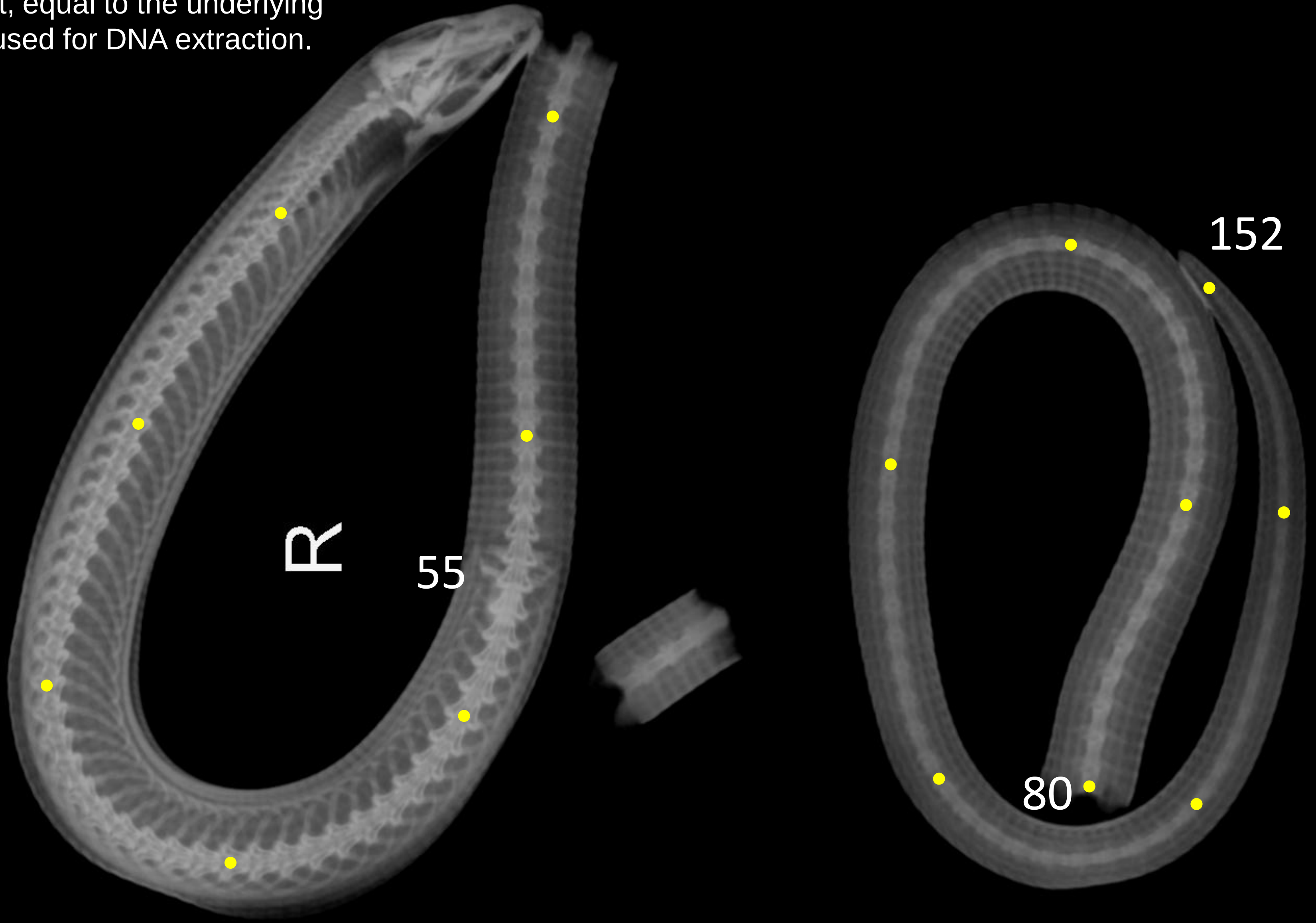

NMNS 14493

Paraneotype, adult male

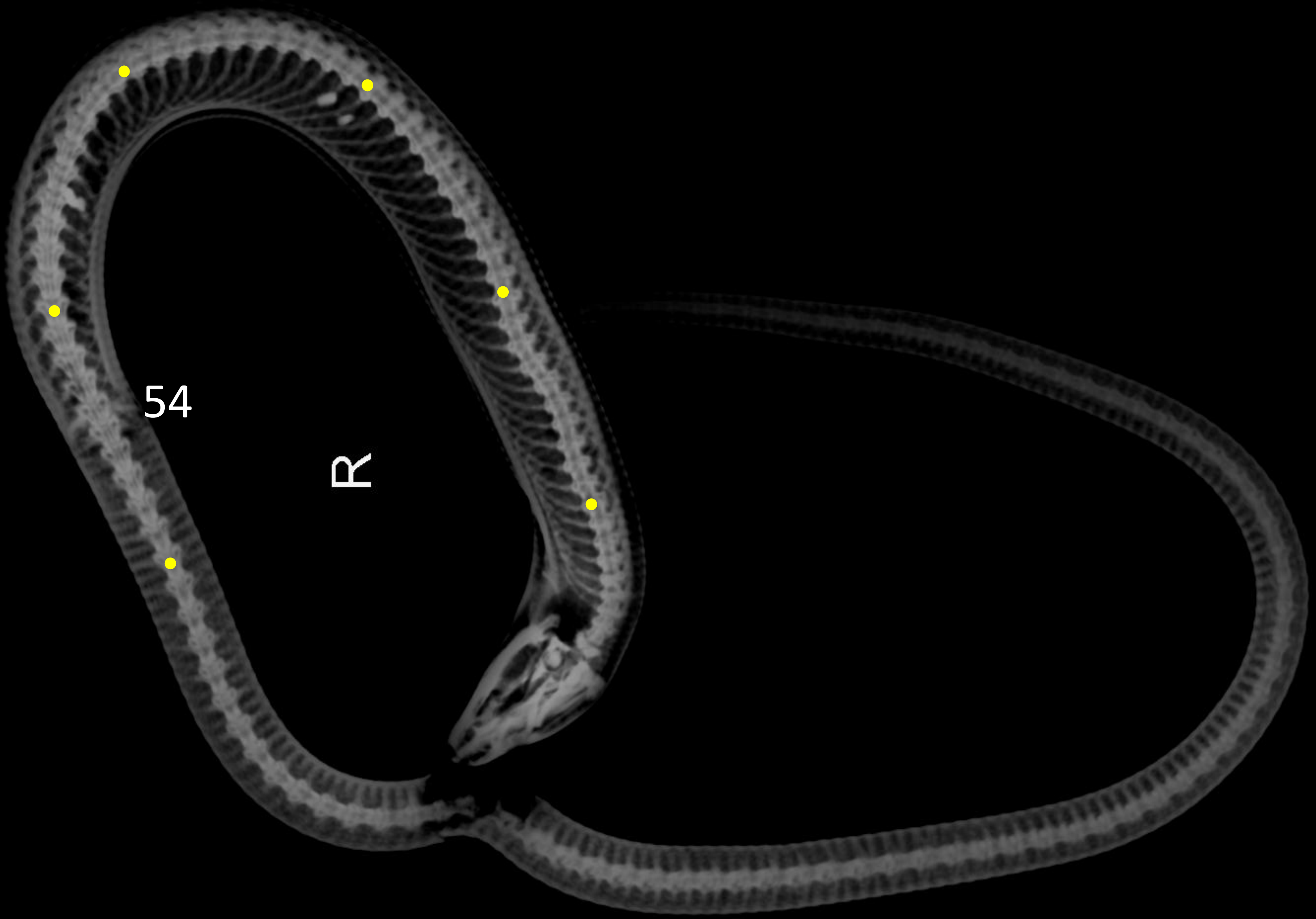

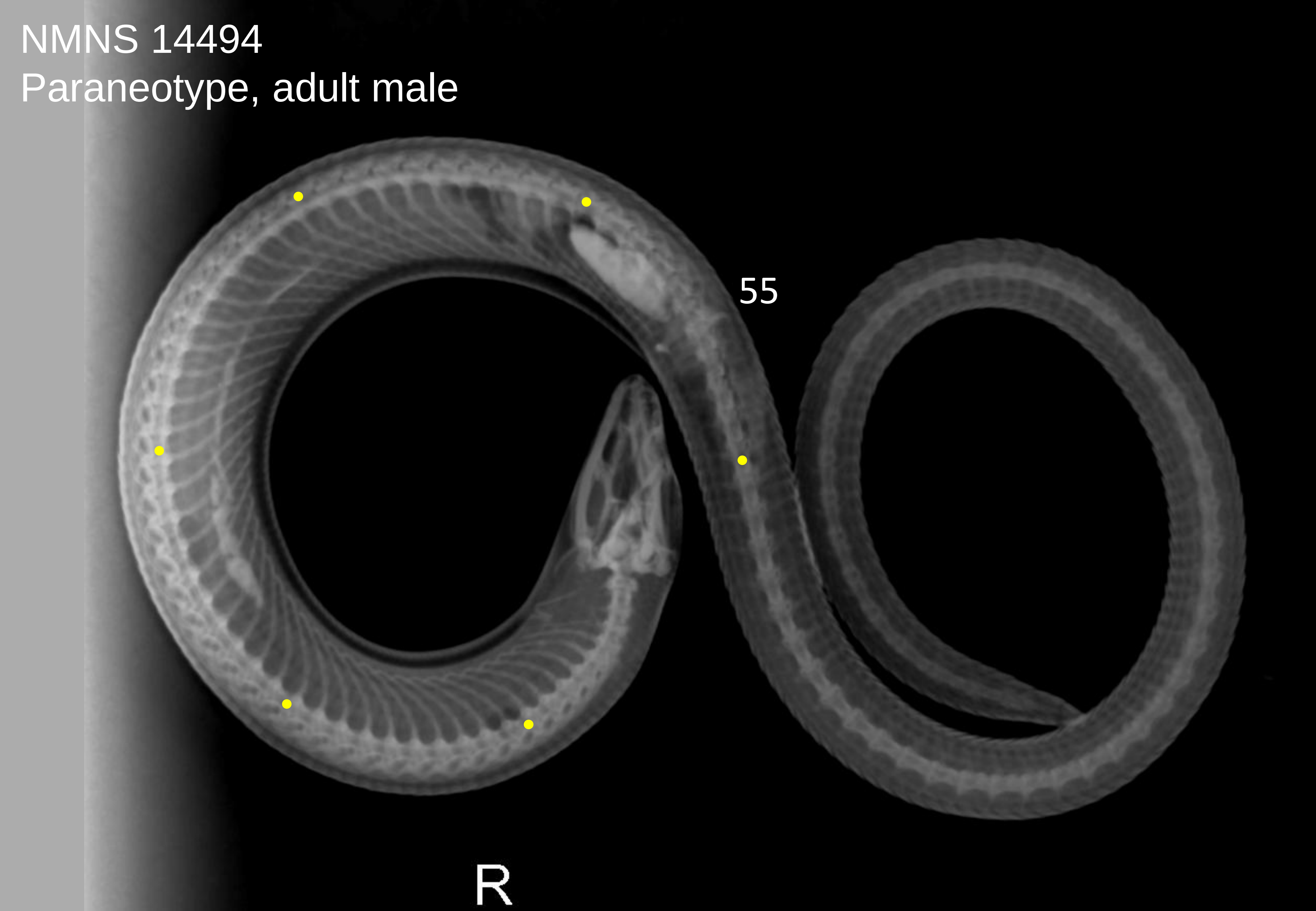

NMNS 14494
Paraneotype, adult male
55
R

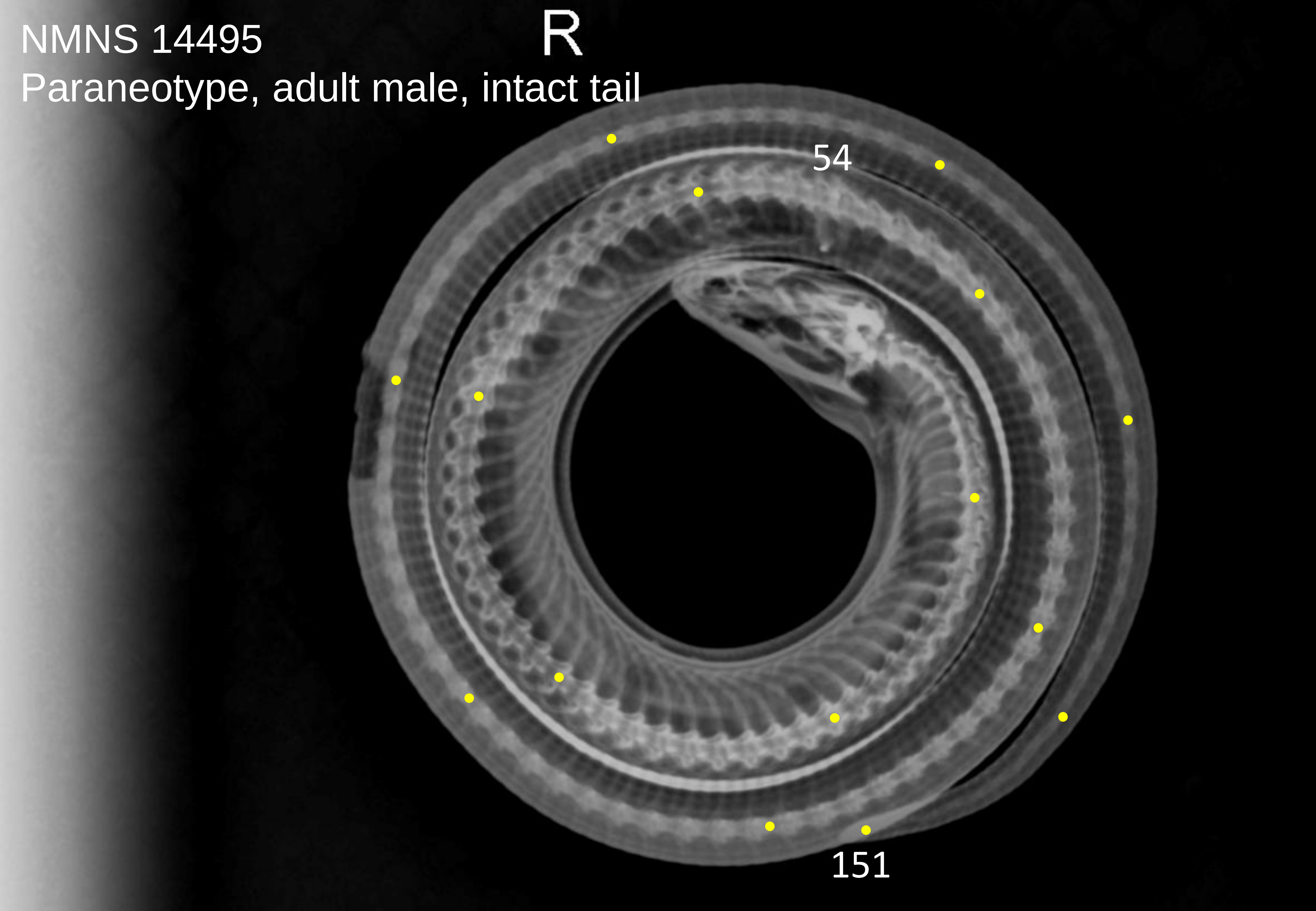
NMNS 14495
Paraneotype, adult male, intact tail
R
54
151

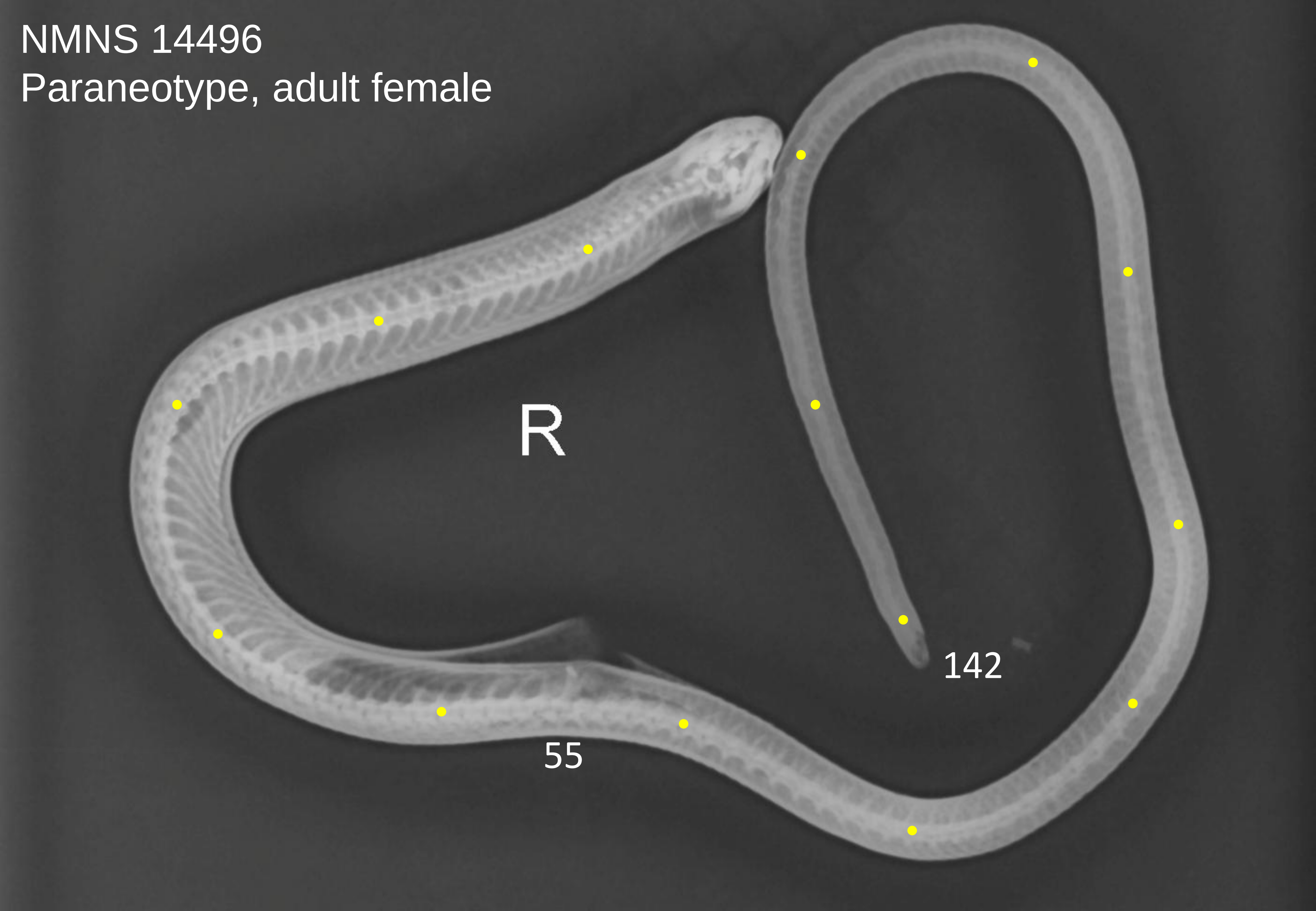
NMNS 14496
Paraneotype, adult female
R
142
55

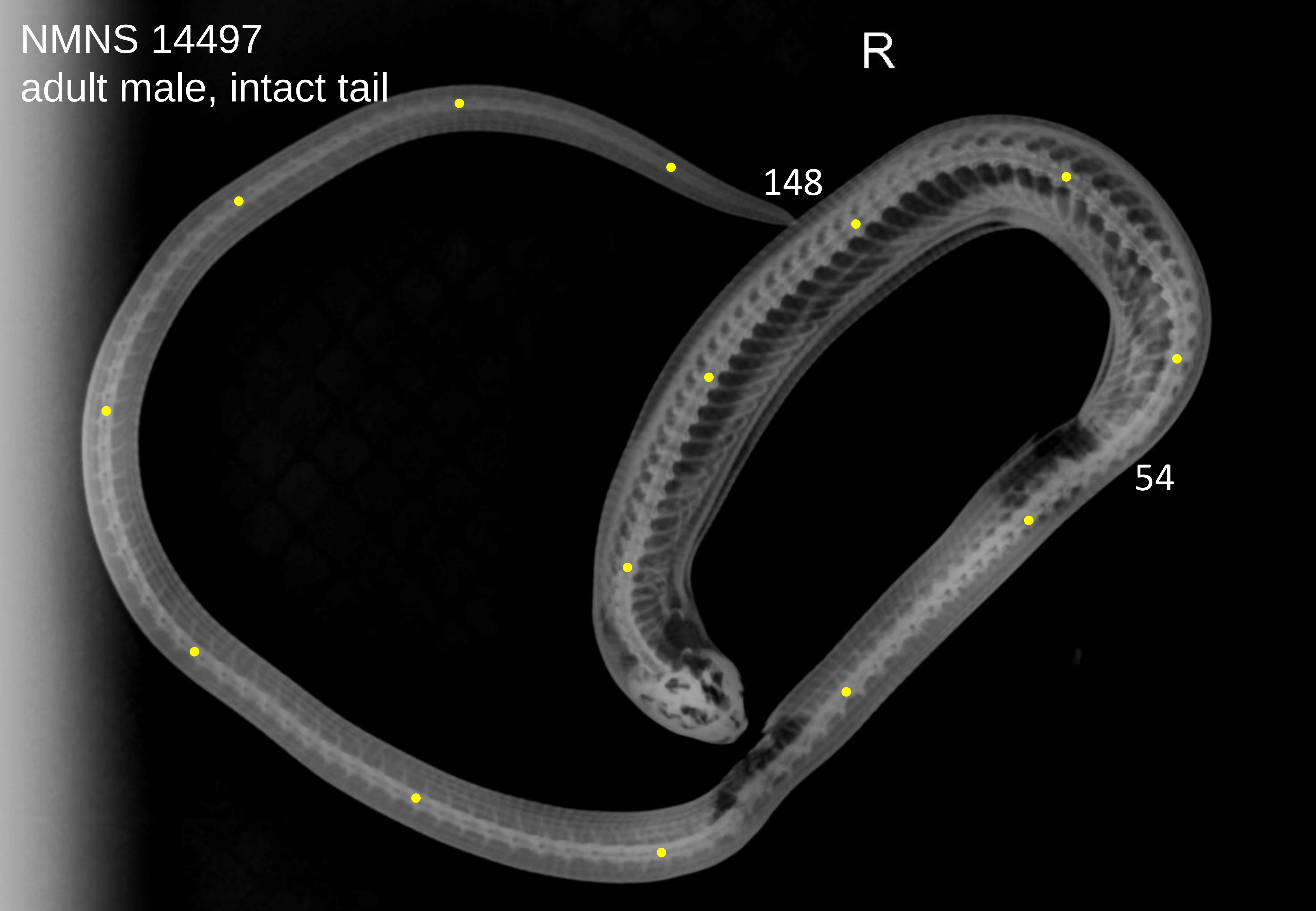

NMNS 14497
adult male, intact tail
R
148
54

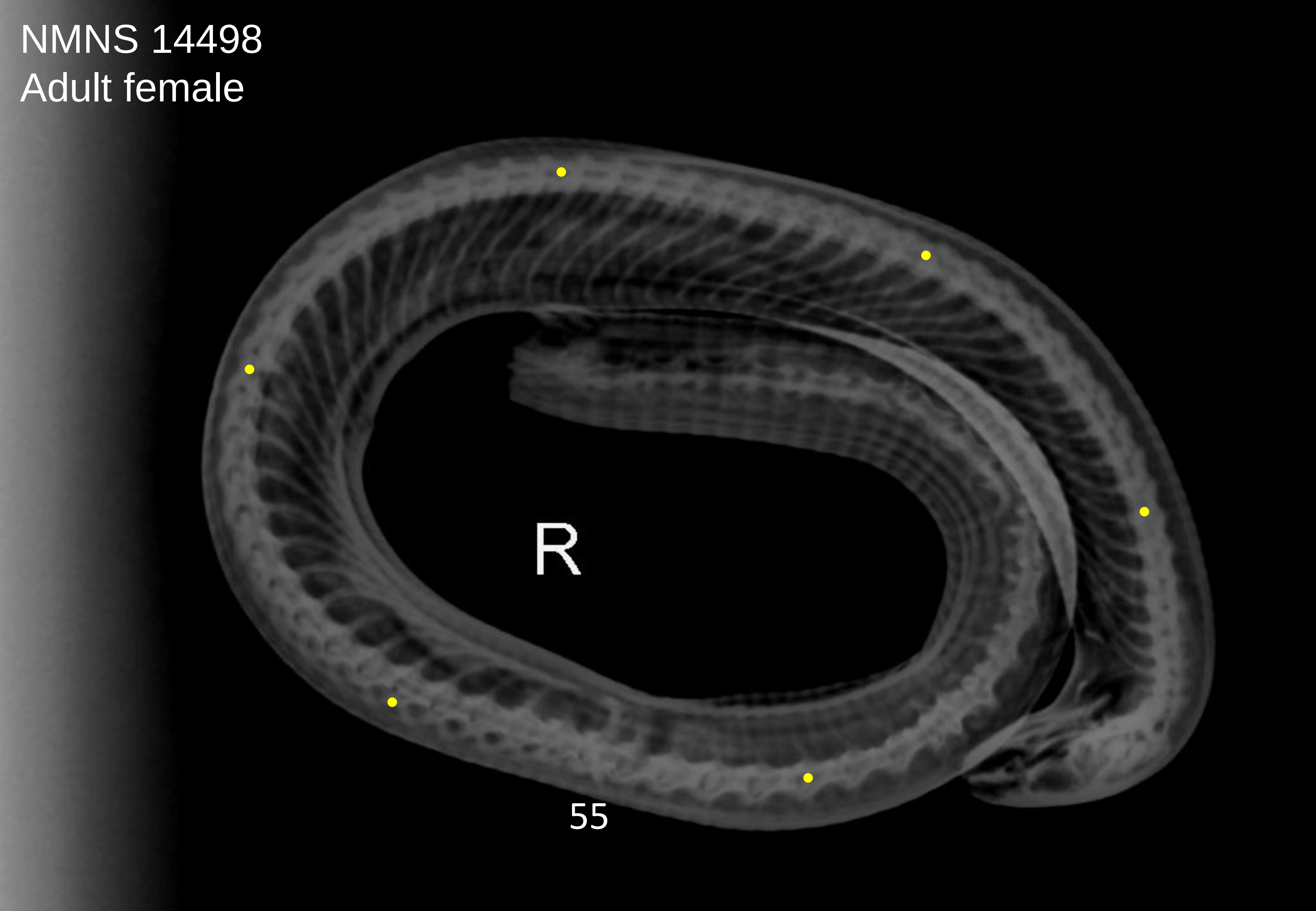

NMNS 14498
Adult female
R
55

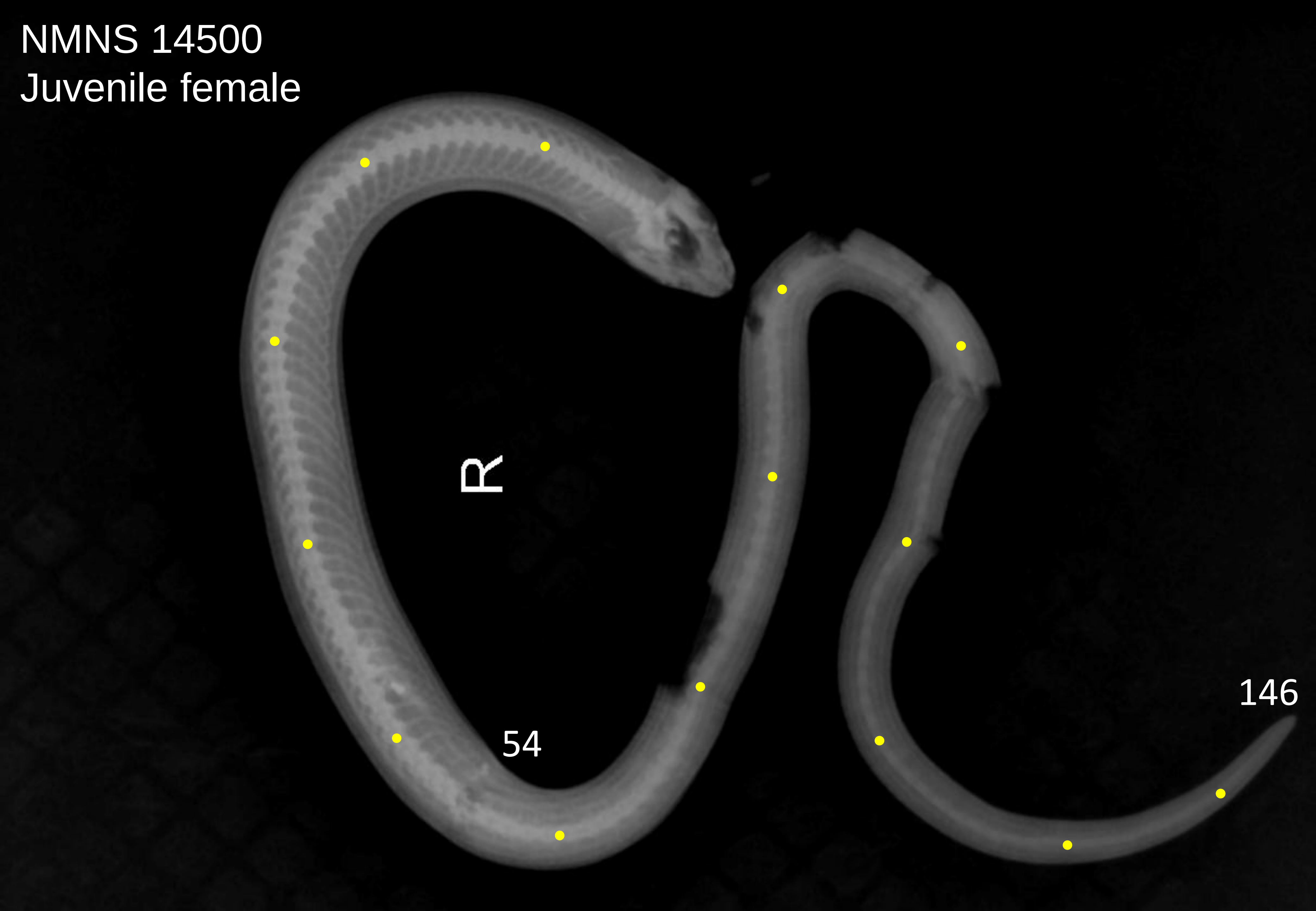
NMNS 14500
Juvenile female
R
54
146

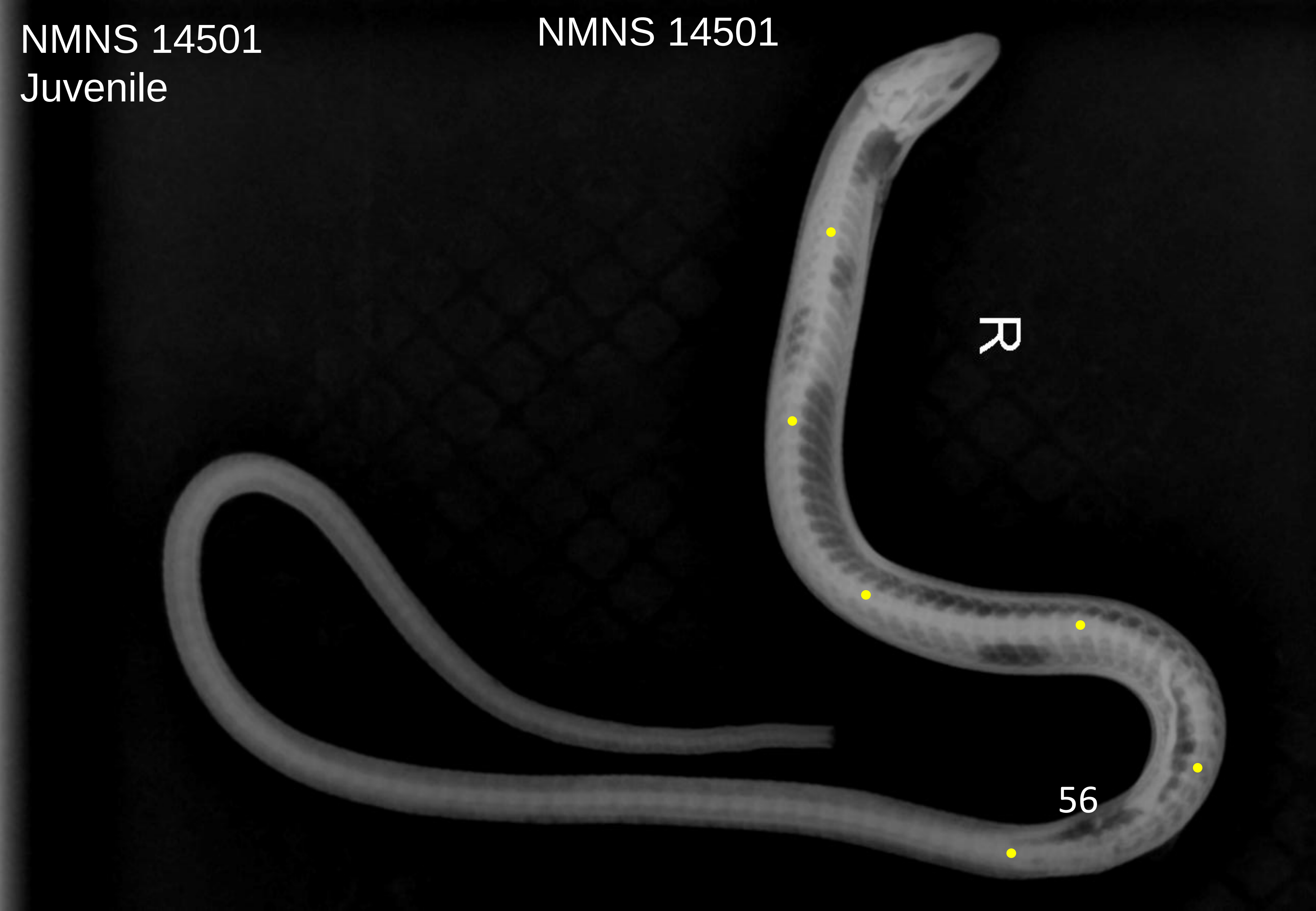
NMNS 14501
Juvenile
NMNS 14501
R
56

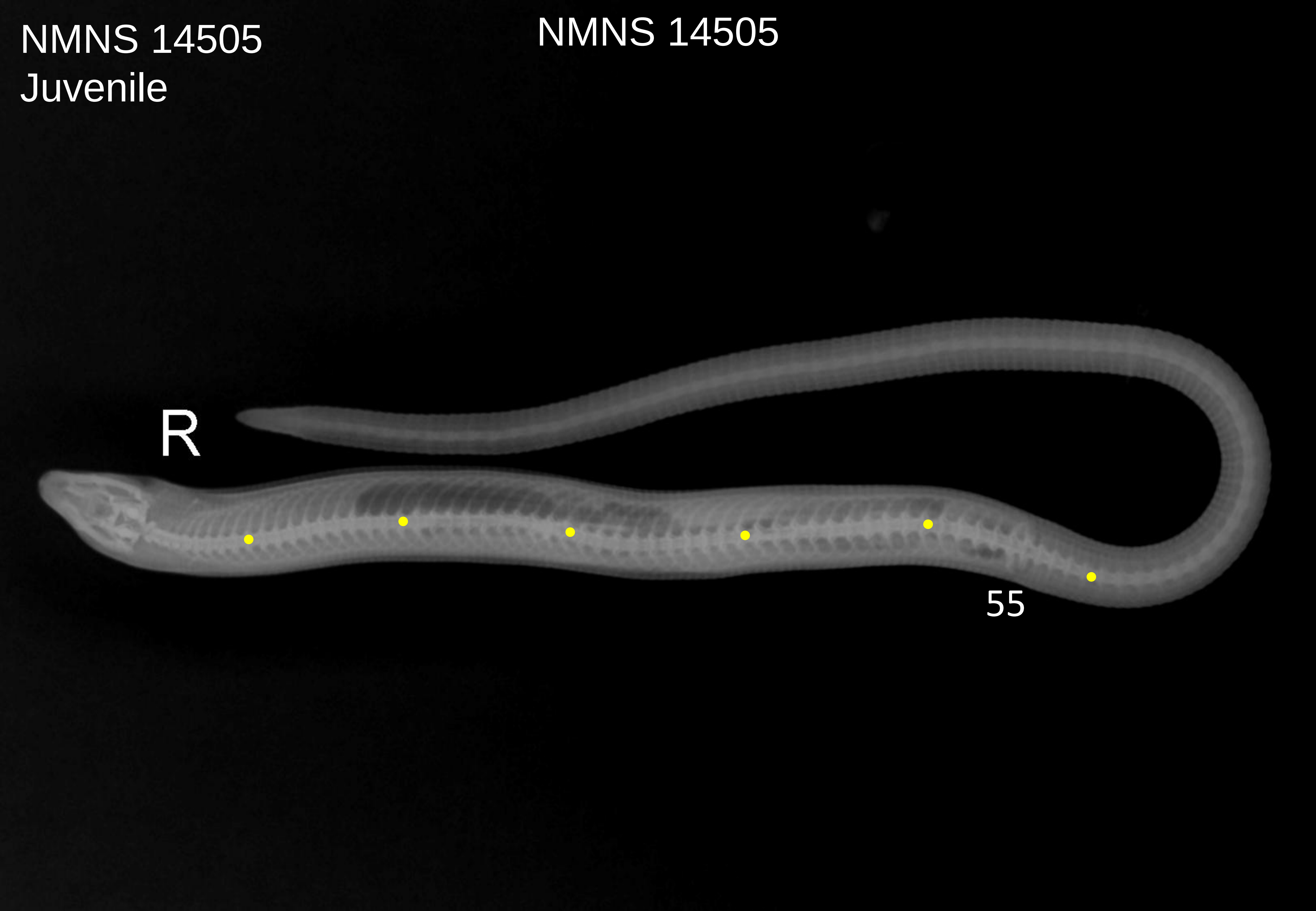
NMNS 14505
Juvenile
NMNS 14505
R
55
